# Supplementary material for: Cost-effectiveness of a pediatric operating room installation in Sub-Saharan Africa
Source: PLOS Glob Public Health. 2024 Mar 27;4(3):e0001748. doi: 10.1371/journal.pgph.0001748 (PMC10971580; doi:10.1371/journal.pgph.0001748)
Supplement: S1 Text — (DOCX) [file pgph.0001748.s003.docx]

**Cost-Effectiveness of a Pediatric Operating Room Installation in Sub-Saharan Africa**

**Supplementary Materials**

**DALY calculations**

The DALY is a measure of disease burden accrued by a patient’s illness and is a combination of years of life lost (YLL) and years lived in disability (YLD). Any public health intervention seeks to prevent or avert the maximal number of DALYs. A single YLL equates to a full DALY, while YLDs are multiplied by a disability weight (DW), which is an unitless value from 0-1 corresponding to the severity of the illness:

*DALYs = YLLs + YLDs*

*YLL = Years of Life Lost*

*YLD = Years Lived with Disability x DW*

When including a time discount *r* and age weight *K*, DALYs are calculated following Fox-Rushby’s method^1^:

Years of life lost (YLL) were calculated by the following formula:

Where:

K – Modulates age weight inclusion (1 or 0)
C – Mathematical Constant (0.1658)
r – Discount rate (0%, 3%, or 6%)
a – Age at death
b - Parameter from age weighting function (0.04)
e – Natural logarithm root (2.72)
L – Life expectancy

When the discount rate is zero:


YLDs were calculated using the following formula where D is the disability weight. In this equation ***a*** is age at onset of disease and ***L*** is number of years lived with the disability:

When the discount rate is equal to zero the formula simplifies to:

Adjustment of YLL to the year of disease onset was calculated using:

*DALY at age x = DALY(y)e^-rs^*

**Sources of Model Inputs in Health Effectiveness and Costs**

*Table A: Disease associated disability weights used in our model to inform health states and effectiveness*

| **Diagnosis** | **DW** | **Source** |
| --- | --- | --- |
| Pre-existing ostomy req. closure | 0.106 | GBD 2019 (Stoma + Disfigurement)^2,3^ |
| ARM - High (initial presentation) | 0.081 | Smith 2018^4^ |
| ARM - High (post ostomy) | 0.351 | Poenaru 2017 (PC-TTO approach, Kenya)^5^ |
| ARM - High (post repair) | 0.209 | GBD 2019 (Stoma + Abdominopelvic problem)^2,3^ |
| ARM - Low | 0.280 | Smith 2018^4^ |
| Vestibular Anus | 0.280 | Smith 2018^4^ |
| Cloaca (initial presentation) | 1.000 | Yap 2018^6^ |
| Cloaca (post colostomy) | 0.351 | Poenaru 2017 (PC-TTO approach, Kenya)^5^ |
| Cloaca (post repair) | 0.209 | GBD 2019 (Stoma + Abdominopelvic problem)^2,3^ |
| Hirschsprung's (with perforation) | 1.000 | GBD 2019 Appendix 1 Table S5^7^ |
| Hirschsprung's (initial presentation) | 0.720 | Smith 2018^4^ |
| Hirshsprung's (post ostomy) | 0.351 | Poenaru 2017 (PC-TTO, approach Kenya)^5^ |
| Hirschsprung's (post repair) | 0.209 | GBD 2019 (Stoma + Abdominopelvic problem)^2,3^ |
| Hirschsprung's (req, biopsy) | 0.720 | Smith 2018^4^ |
| Other Anorectal Disease | 0.280 | Smith 2018^4^ |
| Gastroschisis | 1.000 | Ford 2016^8^ |
| Omphalocele | 0.920 | Poenaru 2015^9^ |
| Cloacal Exstrophy | 1.000 | Yap 2018^6^ |
| Pyloric Stenosis | 1.000 | Ullrich 2020^10^ |
| Duodenal Atresia | 0.758 | Poenaru 2017 (PC-TTO approach, Kenya)^5^ |
| Jejunal Atresia | 0.758 | Poenaru 2017 (PC-TTO approach, Kenya)^5^ |
| Ileal Atresia | 0.758 | Poenaru 2017 (PC-TTO approach, Kenya)^5^ |
| Colonic Atresia | 0.758 | Poenaru 2017 (PC-TTO approach, Kenya)^5^ |
| Biliary Atresia | 1.000 | Ullrich 2020^10^ |
| Choledochal Cyst | 1.000 | Yap 2018^6^ |
| Mesenteric Cyst | 0.114 | GBD 2019 (Abdominopelvic problem, moderate)^2,3^ |
| Thyroglossal Cyst | 0.067 | GBD 2019 (Disfigurement, level 2)^2,3^ |
| Esophageal Atresia | 0.850 | Smith 2018^4^ |
| Congenital Diaphragmatic Hernia | 1.000 | Ullrich 2020^10^ |
| Umbilical or Epigastric hernia | 0.100 | Shillcutt 2010^11^ |
| Inguinal or Scrotal Hernia | 0.096 | Shillcutt 2010, Eeson 2015^11,12^ |
| Hydrocele | 0.100 | Shillcutt 2010^11^ |
| Undescended Testis | 0.220 | Poenaru 2015^9^ |
| Testicular Torsion | 0.324 | GBD 2019 (Abdominopelvic problem severe)^2,3^ |
| Bladder Exstrophy | 0.342 | GBD 2019 (Vesicovaginal fistula)^2,3^ |
| Posterior Urethral Valve | 0.571 | GBD 2019 (End-stage renal disease on dialysis)^2,3^ |
| Hypospadias | 0.275 | Poenaru 2017 (PC-TTO approach, Kenya)^5^ |
| Disorder of Sexual Differentiation | 0.008 | GBD 2019 (Infertility)^2,3^ |
| Cleft Lip or Palate | 0.122 | Smith 2018^4^ |
| Congenital Heart Disease | 0.323 | Smith 2018^4^ |
| Hydrocephalus | 0.740 | Poenaru 2017 (PC-TTO approach, Kenya)^5^ |
| Spina Bifida | 0.593 | Smith 2018^4^ |
| Sacrococcygeal Teratoma | 1.000 | GBD Cancer 2019^13^ |
| Wilms Tumor (Nephroblastoma) | 1.000 | GBD Cancer 2019^13^ |
| Neuroblastoma | 1.000 | Yap 2018^6^ |
| Hepatoblastoma | 1.000 | Yap 2018^6^ |
| Rhabdomyosarcoma | 1.000 | Yap 2018^6^ |
| Intraabdominal Mass or Tumor | 1.000 | GBD Cancer 2019^13^ |
| Ovarian Mass or Tumor | 0.288 | GBD 2019 (Cancer, initial diagnosis)^2,3^ |
| Testicular or Scrotal Mass or Tumor | 0.288 | GBD 2019 (Cancer, initial diagnosis)^2,3^ |
| Soft Tissue Mass or Tumor | 0.067 | GBD 2019 (Disfigurement Level 2)^2,3^ |
| Extremity Mass or Tumor | 0.067 | GBD 2019 (Disfigurement Level 2)^2,3^ |
| Burkitt's Lymphoma | 1.000 | GBD Cancer 2019^13^ |
| Myeloproliferative Disorder | 1.000 | GBD Cancer 2019^13^ |
| Intussusception | 0.800 | Smith 2018^4^ |
| Malrotation or Volvulus | 0.114 | GBD 2019 (Abdominopelvic problem, moderate)^2,3^ |
| Small Bowel Obstruction | 0.800 | Smith 2018^4^ |
| Constipation or Fecal Impaction | 0.114 | GBD 2019 (Abdominopelvic problem, moderate)^2,3^ |
| Rectal Prolapse | 0.188 | GBD 2019 (Disfigurement Level 2 with pain)^2,3^ |
| Blunt Trauma | 1.000 | GBD 2019 Appendix 1 Table S5^7^ |
| Splenic Injury | 1.000 | GBD 2019 Appendix 1 Table S5^7^ |
| Penetrating Trauma | 1.000 | GBD 2019 Appendix 1 Table S5^7^ |
| Burns | 0.455 | GBD 2019 (>20% body burn without treatment)^2,3^ |
| Other Trauma | 0.250 | GBD 2019, Mathers 2004^2,3,14^ |
| Non-necrotizing Skin & Soft Tissue Infection | 0.133 | GBD 2019 (Infectious disease acute) |
| Necrotizing Soft Tissue Infection | 1.000 | GBD 2019 Appendix 1 Table S5^7^ |
| Appendicitis | 0.324 | GBD 2019 (Abdominopelvic problem, severe)^2,3^ |
| Primary Peritonitis | 1.000 | GBD 2019 Appendix 1 Table S5^7^ |
| Typhoid Ileal Perforation | 0.800 | Smith 2018^4^ |
| Other Intestinal Perforation | 0.800 | Smith 2018^4^ |
| Cholecystitis or Cholangitis | 0.324 | GBD 2019 (Abdominopelvic problem, severe)^2,3^ |
| Necrotizing Enterocolitis | 1.000 | Ullrich 2020 |
| Neonatal Sepsis | 1.000 | GBD 2019 Appendix 1 Table S5^7^ |
| Thoracic Empyema | 0.408 | GBD 2019 (severe respiratory illness, chronic)^2,3^ |
| Chronic Osteomyelitis | 0.219 | GBD 2019 (Infectious disease)^2,3^ |
| Post-Surgery Complication | 0.133 | GBD 2019 (Infectious disease)^2,3^ |
| Gastroesophageal Reflux Disease | 0.011 | GBD 2019 (Dysphagia from reflux)^2,3^ |
| Conjoint Twins | 0.237 | GBD 2019 (Disfigurement level 2)^2,3^ |
| Circumcision conditions | 0.114 | Chatterjee 2015, GBD 2019^2,3,15^ |
| Foreign Body in the Airway | 1.000 | GBD 2019 Appendix 1 Table S5^7^ |
| Adenoid or Tonsillar Enlargement | 0.019 | GBD 2019 (Chronic respiratory problems, mild)^2,3^ |
| Laryngeal Papilloma | 0.019 | GBD 2019 (Chronic respiratory problems, mild)^2,3^ |
| Esophageal Stricture | 0.114 | GBD 2019 (Abdominopelvic problem, moderate)^2,3^ |
| Hypersplenism | 0.011 | GBD 2019 (Abdominopelvic problem, mild)^2,3^ |
| Chordee | 0.114 | Chatterjee 2015, GBD 2019^2,3,15^ |
| Meatal Stenosis | 0.114 | Chatterjee 2015, GBD 2019^2,3,15^ |
| Cholelithiasis or Biliary Colic | 0.011 | GBD 2019 (Abdominopelvic problem, mild)^2,3^ |

*Table B: List of long-term reusable and disposable operating room equipment donated to inform the charity perspective with their associated life time. AHA = American Hospital Association^16^, BEAG = Biomedical Engineering Advisory Group Guidance Paper 2004^17^*

|  | |  | |  | | |
| --- | --- | --- | --- | --- | --- | --- |
| **EQUIPMENT** | **Supplier** (company) | | **Life** (years) | | **Lifespan Source** | **#** |
| Laryngoscope sets (1 McIntosh + 1 Miller) | [Longlife Surgical](http://www.longlife.com.pk/laryngoscopes.php) | | 10 | | Sherman 2018^18^ | 3 |
| 2.5x Magnification Loupes | [KEELER](https://support.keeler-global.com/assets/Keeler%20Warranty%20Website.pdf) | | 3 | | 3-year warranty | 1 |
| Stethoscope | [NARANG](https://www.narang.com/diagnostic-equipments-products/stethoscopes-spare-parts/index.php) | | 2 | | Projected lifespan of 2 years | 2 |
| Surgical Instrument Kit | [MAHR SURGICAL](https://www.mahrsurgical.co.uk/product-category/instruments/) | | 5 | | Leiden 2020^19^ | 1 |
| Operating Table | Generic | | 15 | | AHA 2018 "Table; operating"  BEAG 2004 "Table” | 1 |
| Patient Stretcher | [Formed](http://formed.eu.pl/en/transport-stretchers/33/lama-transport-stretchers.html) | | 7 | | AHA 2018 "Stretcher; Hydraulic"  BEAG 2004 "Strechers; portable" | 3 |
| Patient Privacy Screen | [BRISTOL MAID](https://www.healthandcare.co.uk/screens-mirrors/bristol-maid-three-section-solid-privacy-screen.html) | | 1 | | 12-month warranty | 1 |
| Emergency Trolley | [BRISTOL MAID](https://www.medicalexpo.com/product-manufacturer/bristol-maid-stainless-steel-trolley-4940-254.html) | | 10 | | AHA 2018 "Cart; emergency-isolation" | 1 |
| Anaesthetic Trolley | [BRISTOL MAID](https://www.medicalexpo.com/product-manufacturer/bristol-maid-stainless-steel-trolley-4940-254.html) | | 10 | | AHA 2018 "Cart; Medicine" | 1 |
| Instrument Trolley | [BRISTOL MAID](https://www.medicalexpo.com/product-manufacturer/bristol-maid-stainless-steel-trolley-4940-254.html) | | 10 | | AHA 2018 "Cart; Supply" | 2 |
| Mayo Trolley | [BRISTOL MAID](https://www.medicalexpo.com/product-manufacturer/bristol-maid-stainless-steel-trolley-4940-254.html) | | 15 | | AHA 2018 "Stand; Mayo" | 2 |
| Contamination Tub and Trolley | [BRISTOL MAID](https://www.medicalexpo.com/product-manufacturer/bristol-maid-stainless-steel-trolley-4940-254.html) | | 15 | | AHA 2018 "Stand; Basin" | 2 |
| Dirty Linen Trolley | [BRISTOL MAID](https://www.medicalexpo.com/prod/bristol-maid-hospital-metalcraft/product-67911-481070.html) | | 10 | | AHA 2018 "Cart; Linen" | 2 |
| Saline / Drip Stand Bundle | Generic | | 15 | | AHA 2018 "Stand; Intravenous" | 4 |
| Bowl Stand Bundle | Generic | | 15 | | AHA 2018 "Stand; Basin" | 2 |
| Revolving Stool | [SEERS MEDICAL](https://www.seersmedical.com/en-us/product/basic-operators-stool) | | 5 | | 5-year warranty | 2 |
| Kick Basin Bundle | Generic | | 15 | | AHA 2018 "Bin - metal or wood" | 2 |
| Surgeons Step Stool/Foot Stool | Generic | | 15 | | AHA 2018 "Operating stool" | 1 |
| Cidex Tray | Generic | | 7 | | AHA 2018 "Steris sterilizing system"  BEAG "Sterilization packaging" | 4 |
| Gratnell Storage Trolley (for Diathermy Unit) | [GRATNELLS](https://www.gratnells.com/storage-trolleys) | | 10 | | AHA 2018 "Cart; Supply" | 1 |
| Mesh Shelving | [SHOPFITTING WAREHOUSE](https://www.shopfittingwarehouse.co.uk/shelving-racking/grid-mesh-shelving/shelves-accessories) | | 20 | | AHA 2018 "Shelving - portable - steel" | 2 |
| Castor Wheels for Mesh Shelving (set of 4) | [SHOPFITTING WAREHOUSE](https://www.shopfittingwarehouse.co.uk/shelving-racking/grid-mesh-shelving/shelves-accessories) | | 20 | | AHA 2018 "Shelving - portable - steel" | 1 |
| Metal Lockable Cabinet | [SILVERLINE](https://www.directofficesupply.co.uk/4-drawer-lockable-filing-cabinet-p116091) | | 5 | | 5-year guarantee | 1 |
| Kidney Tray and lotioon bowl bundle | [NARANG](https://www.narang.com/hospital-holloware/kidney-trays-stainless-steel/index.php) | | 3 | | AHA 2018 "Surgical instruments" | 1 |
| Mobile Operating Light Bundle | [Bender](https://www.bender-uk.com/products/operating-lights/q-flowtm-mobile-operating-light) | | 5 | | 5-year warranty for all equipment | 1 |
| Warming Blanket, control unit (W-300) & support bracket 110V | [MEDWARM](https://www.sbtmedical.co/en/products/general-products/patient-warming-system/item/66-medwarm-adult-and-pedicatric-blankets.html) | | 2 | | 2-year warranty period | 1 |
| A3 Lightbox/X-ray Viewer - (Model No:4040) | [OYPLA](https://oypla.com/sports-leisure/arts-crafts-hobbies/a3-light-box-drawing-board-tracing-copy-adjustable-brightness) | | 1 | | 1-year warranty | 1 |
| Small Battery Suction Unit | [MGE](https://www.mgeworldwide.com/sam-eps) | | 2 | | 2-year warranty | 1 |
| Large Suction Unit (inc. accs) | [MGE](https://www.mgeworldwide.com/sam-eps) | | 2 | | 2-year warranty | 1 |
| Defibrillator (Zoll AED 3) + pad | [ZOLL](https://www.zoll.com/products/defibrillators) | | 5 | | AHA 2018 "Defibrillator"  BEAG "Defibrilator paddle pads" | 1 |
| Oxygen Concentrator (5L) Drrive 240V | [Drive Devilbiss](https://www.drivemedical.com/us/en/products/respiratory/oxygen-therapy-%26-accessories/oxygen-therapy/devilbiss-5-liter-oxygen-concentrator/p/525DS/downloadPdf) | | 3 | | 5-year extended warranty | 2 |
| Anaesthetic Machine (WATO EX-35) | [MINDRAY](http://mindray.sy/wp-content/uploads/2017/08/new-WATO-EX-35-Brochure-20160226.pdf) | | 7 | | AHA 2018 "Anesthesia unit"  BEAG "Anaesthesia unit absorbers" | 1 |
| Breathing accessories | [Intersurgical](https://www.intersurgical.com/products/anaesthesia/breathing-system-accessories) | | 7 | | BEAG 2004 "Breathing circuits - oxygen administration" | 1 |
| Lifebox Patient Monitor/Pulse Oximeter | [ACARE](https://www.lifebox.org/purchase-oximeter/) | | 2 | | 2-year warranty according to Lifebox | 2 |
| Vaporiser | [PENLON](https://www.penlon.com/Product-Groups/Vaporizer/Sigma-Delta) | | 10 | | BEAG 2004 "Anaesthesia unit vaporizers" | 2 |
| Blood and Infusion Warmer | Generic | | 7 | | AHA 2018 "Blood warmer" | 1 |
| Electrosurgical Unit/Diathermy (A1250S) | [BOVIE (SH)](https://shop.symmetrysurgical.com/en/product/portfolios-electrosurgery-generators/A1250S) | | 4 | | 4-year warranty | 1 |
| Electrosurgical Accessories Bundle | [GRAZEDEAN LTD](http://www.grazedean.com/) | | 7 | | BEAG 2004 "Forceps - electrosurgical" | 1 |
| Advanced Patient Monitor (EPM12), AGM module & accessories kit | [MINDRAY](https://www.mindraynorthamerica.com/patient-monitoring-systems/monitors/epm-10m-12m/) | | 3 | | 3-year warranty | 1 |
| Advanced Patient Monitor (iMEC 12) & accessories kit | [MINDRAY](http://mindray.sy/wp-content/uploads/2019/10/iMEC_Operators-Manual.pdf) | | 3 | | Product life of 3 years | 2 |
| Advanced Patient Monitor Stand | MINDRAY | | 10 | | AHA 2018 "Patient monitoring equipment" | 2 |
| Accessories Spares Bundle (Monitor Extras) | MINDRAY | | 3 | | See above for Patient Monitors | 1 |
| Headlight (battery operated LED) | [LEDLENSER](https://www.ledlenserusa.com/collections/headlamps/products/h5-core) | | 7 | | 7-year warranty registering online | 1 |
| Smart Battery charger | [EBL OFFICIAL](https://www.amazon.com/EBL-Battery-Charger-Rechargeable-Technology/dp/B01D9TUL8Y) | | 5 | | AHA 2018 "Battery charger" | 1 |
| Organ & Tissue Medical Scales | [SECA](https://www.seca.com/fileadmin/documents/product_sheet/seca_pst_856_en-gb.pdf) | | 8 | | 8-year warranty | 1 |
| Nailbrush | [NARANG](https://www.narang.com/misc-surgical-medical-products/nail-brushes/index.php) | | 3 | | Recorded shelf life | 100 |
| Armboard IV Splints Reusable | NARANG | | 3 | | Recorded shelf life | 10 |
| Artificial Resuscitator Bundle | [NARANG](https://www.narang.com/anaesthesia-equipments-products/silicone-autoclavable/AN220.php) | | 10 | | AHA 2018 "Resuscitator"  BEAG 2004 "Resuscitators - pulmonary" | 15 |
| Bundle Patient Identification Bands | [NARANG](https://www.narang.com/hospital-scrubs-linens-manufacturers/hospital-bracelets-patient-wristband/DS655.php) | | 0.5 | | 400 cases / year | 200 |
| Protective Safety Goggles | [3M](https://www.3m.com/3M/en_US/p/c/ppe/eye-protection/goggles/i/safety/personal-safety/) | | 1 | | 12-month warranty in similar company | 6 |
| Box of Face Masks | [PPE EXTRA](https://www.ppextra.com/?v=7516fd43adaa) | | 0.125 | | 400 cases / year * 4 masks per case | 200 |
| Box of Disposable Drape Sheets 60x60 | [NARANG](https://www.narang.com/medical-disposables/surgical-drape-sets-orange/index.php) | | 0.25 | | 400 cases per year / 100x | 100 |
| Cotton Drape Sheet 88x90 | [NARANG](https://www.narang.com/hospital-scrubs-linens-manufacturers/hospital-linens/DS638.php) | | 6 | | 100 * 75 washes = 400 cases/year * 3 drapes/case * 6 years | 100 |
| Surgical Gowns Small | N/A | | 1 | | 75 washes per gown, x 25 gowns ~ 400 cases per year * 4 gowns used per case (McQuerry 2021)^20^ | 25 |
| Surgical Gowns Medium | Clean room supplies | | 1 | | McQuerry 2021^20^ | 25 |
| Surgical Gowns Large | Clean room supplies | | 1 | | McQuerry 2021^20^ | 25 |
| Surgical Gowns XL | Clean room supplies | | 1 | | McQuerry 2021^20^ | 25 |

*Patient Cost Perspective: Out-of-pocket (OOP) costs were derived from the perioperative patient database, obtained from an in-patient economic survey conducted at the time of the patients’ surgical admission. The following OOP costs per patient informed the randomized distribution of the probabilistic sensitivity analysis.*

**Median OOP cost:** $79

**5^th^ %ile OOP cost:** $13

**95^th^ %ile OOP cost:** $550

*Table C: Cost of disposable equipment used on a case-by-case basis for each surgery, financed by the hospital system.*

| **DISPOSABLE EQUIPMENT** | **Count per case** | **US Dollars 2021** | **Nigerian Naira 2021** |
| --- | --- | --- | --- |
| Airway suction catheter | 1 | $0.76 | 110 |
| Gauze roll piece | 4 | $4.44 | 640 |
| Gauze pack | 1 | $19.93 | 2875 |
| Cotton wool roll | 1 | $6.79 | 980 |
| Endotracheal tube, plastic, cuffed | 1 | $3.40 | 490 |
| Saline syringes | 3 | $7.62 | 1100 |
| IV cannula and needle | 1 | $0.91 | 132 |
| Latex gloves single, non-sterile | 6 | $0.55 | 80 |
| Latex gloves pair, sterile | 3 | $24.95 | 3600 |
| Hibiscrub 4% bottle | 0.005 | $415.89 | 60000 |
| Providine Iodine 10% bottle | 0.0125 | $41.59 | 6000 |
| Nylon suture with needle | 2 | $10.40 | 1500 |
| Absorbable suture with needle | 2 | $10.40 | 1500 |
| Scalpel blade size 11 | 1 | $11.78 | 1700 |
| Scalpel blade size 15 | 1 | $11.78 | 1700 |
| Autoclave tape | 0.02 | $24.26 | 3500 |
| Foley catheter and bag* | 1 | $3.47 | 500 |
| Colostomy bag* | 1 | $6.93 | 1000 |
| Gastrostomy tube* | 1 | $3.47 | 500 |
| Nasogastric tube* | 1 | $0.51 | 74 |

*Used only in certain cases depending on the necessity

*Table D: Cost of common perioperative medication costs from the hospital pharmacy included into the model.*

| **MEDICATION (Unit)** | **Nigerian Naira (NGN)** | **Units** | **NGN/Unit** | **$/Unit** | **Unit/kg** |
| --- | --- | --- | --- | --- | --- |
| Isoflurane (ml) | 50000 | 250 | 200 | $1.3863 | 30 |
| Bupivicaine (mg) | 1850 | 25 | 74 | $0.5129 | 1.5 |
| Paracetamol (mg) | 40 | 25 | 1.6 | $0.0111 | 10 |
| Diclofenac IV (mg) | 440 | 75 | 5.87 | $0.0407 | 0.5 |
| Ketorolac IV (mg) | 0 | 0 | 0 | $0.0000 | 2 |
| Atropine (mg) | 365 | 7 | 52.1 | $0.3614 | 0.01 |
| Ketamine (mg) | 3900 | 50 | 78 | $0.5407 | 2 |
| Thiopental (mg) | 2000 | 1000 | 2 | $0.0139 | 4 |
| Succinylcholine (mg) | 600 | 100 | 6 | $0.0416 | 2 |
| Hydrocortisone (mg) | 300 | 100 | 3 | $0.0208 | 10 |
| Dexameth (mg) | 40 | 4 | 10 | $0.0693 | 10 |
| Propofol (mg) | 2000 | 20 | 100 | $0.6931 | 4 |
| Lidocaine IV (mg) | 350 | 100 | 3.5 | $0.0243 | 2 |
| Tramadol (mg) | 150 | 100 | 1.5 | $0.0104 | 0.1 |
| Atracurium | 1850 | 25 | 74 | $0.5129 | 0.4 |
| Fentanyl (mcg) | 4500 | 100 | 45 | $0.3119 | 2 |
| Amoxicillin (mg) | 155 | 500 | 0.31 | $0.0021 | 100 |
| Ceftriaxone (mg) | 170 | 1000 | 0.17 | $0.0012 | 100 |
| Flagyl (mg) | 170 | 500 | 0.34 | $0.0024 | 15 |

*Table E: Public salary scales that informed the wages of the perioperative staff involved*

| **Designation** | **Position** | **Minimum monthly**  **(NGN 2021)** | **Maximum monthly**  **(NGN 2021)** | **Average monthly (NGN 2021)** | **Average annual**  **(USD 2021 PPP)** |
| --- | --- | --- | --- | --- | --- |
| Consultant (senior) | Pediatric surgeon | 600000 | 900000 | 750000 | 62383 |
| Consultant (senior) | Pediatric anesthetist | 600000 | 900000 | 750000 | 62383 |
| Consultant (junior) | General surgeon | 450000 | 650000 | 550000 | 45748 |
| Consultant (junior) | Anesthetist | 450000 | 650000 | 550000 | 45748 |
| Resident (senior) | Pediatric fellow | 260000 | 350000 | 305000 | 25369 |
| Resident (junior) | Resident (surgery) | 220000 | 270000 | 245000 | 20378 |
| Resident (junior) | Resident (anesthesia) | 220000 | 270000 | 245000 | 20378 |
| Medical officers | Medical officer | 180000 | 600000 | 390000 | 32439 |
| Senior nurse | Senior nurse | 100000 | 150000 | 125000 | 10397 |
| Entry level nurse | Nurse | 50000 | 80000 | 65000 | 5407 |

Salary scales were derived from the following sources and verified by the local perioperative staff:

- <https://www.mysalaryscale.com/blog/nigerian-civil-service-salary-structure/>
- <https://nigerianprice.com/doctors-salary-in-nigeria/>
- <https://facts36.com/salaries-of-doctors-in-nigeria/>
- <https://nigerianprice.com/nurses-salary-in-nigeria/>

Calculations and data sources for the personnel costs attributable to the incremental cases in the incremental arm were as follows:

- OR staff member presence in cases: Staffing for case coverage was collected using the perioperative REDCap patient registry, which provides data on who was present at each case. Therefore, for the extent of the study period, we had case-specific information on which OR staff members covered the case. For example, the perioperative registry informed us that a pediatric anesthesiologist was present in 7.8% of all cases within the study period. To make this proportion applicable to capturing just the “incremental” cases that were performed in the interventional arm, we multiplied the staff member’s % of case presence by the proportion of additional cases that was performed after OR installation. In the base case analysis, this was 34.3%. Therefore, the % of case presence that was attributable to the incremental cases for a pediatric anesthesiologist was 7.8% * 34.3% = 2.7%.
- Staff member salary scales: Personnel salaries were informed by publicly available salary scales set by the Nigerian government and corroborated by the OR staff members at the study hospital. As the salary scales are typically reported in ranges, the midway point was used as the base rate. Using the same example, the pediatric anesthesiologist’s average annual salary in 2021 USD was 45,747.
- Personnel salary attributable to the increased caseload: We calculated this number by multiplying each OR staff member’s annual salary by their proportion of case presence in the incremental number of cases attributable after OR installation. Therefore, following the example of the pediatric anesthesiologist, the attributable salary for the number of cases performed was 45,747 * 2.7% = 1,682.
- Probabilistic sensitivity analysis: The salary scales upper and lower limits were used as the upper and lower bounds of the (gamma) probability distribution. This was the uncertainty factor that we randomized during the Monte Carlo simulation. The other variables that went into calculating the salary scales stayed constant.

*Table F: Per diem hospital in-patient stay costs as informed by the WHO-CHOICE tool 2010.*^21^ *Costs represent only the “hotel” component of hospital costs, excluding drugs and diagnostic tests but including personnel, capital, and food costs.*^22^ *Note that these values were subsequently inflated to 2021 USD (cumulative inflation rate of 25.46%).*

**Designation Currency Average Cost Lower Bound Upper Bound**

Tertiary Hospital in Nigeria 2010 USD, PPP $29.24 $11.27 $64.69

*Fig A: Pie chart showing the relative distributions of the case frequencies over disease categories of the patients treated at Abuja National Hospital within the study period.*


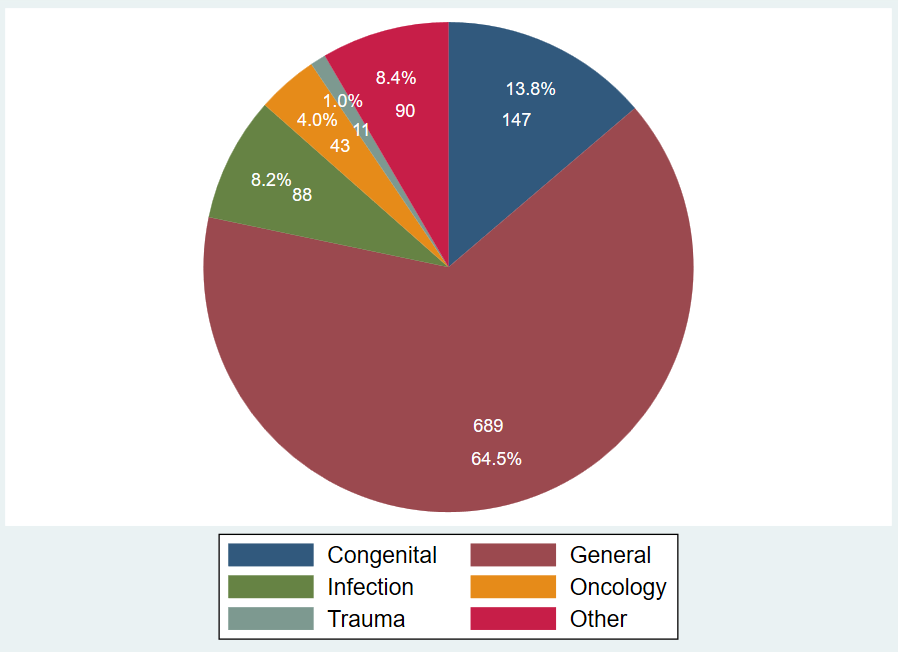


*Table G: Table depicting the top 10 most common cases performed at Abuja National Hospital within the study period, comprising a cumulative 58.8% of the total cases.*

| Procedure | N | % |
| --- | --- | --- |
| Circumcision | 359 | 33.61 |
| Inguinal Hernia Repair | 77 | 7.21 |
| Umbilical/Epigastric Hernia Repair | 42 | 3.93 |
| Appendectomy | 31 | 2.90 |
| Orchiopexy | 30 | 2.81 |
| Hypospadias Reconstruction | 27 | 2.53 |
| Hydrocelectomy | 19 | 1.78 |
| Laparotomy | 17 | 1.59 |
| Intussusception Reduction | 14 | 1.31 |
| Ventriculoperitoneal Shunt | 12 | 1.12 |

*Table H: Catalog of sources and respective ICER values informing figure 4 of the main manuscript to compare our study’s ICER with a wide range of public health interventions.*

| **Study Source** | **Intervention** | **Location** | **ICER (reported year)** | **ICER (2021 USD)** |
| --- | --- | --- | --- | --- |
| Yap 2021^6^ | Pediatric OR installation | Uganda | 80 | 94 |
| This study | Pediatric OR installation | Nigeria | 330 | 330 |
| Source: Saxton 2016^23^ |  |  | **ICER (2015 USD)** | **ICER (2021 USD)** |
| Jha 1998 | Pediatric General Surgery | Guinea | 58 | 68 |
| Jha 1998 | Pediatric General Surgery | Guinea | 119 | 140 |
| Shillcutt 2010 | Pediatric General Surgery | Ghana | 14 | 16 |
| Shillcutt 2013 | Pediatric General Surgery | Ecuador | 103 | 121 |
| Eason 2015 | Pediatric General Surgery | Uganda | 4 | 5 |
| Evans 1996 | Trichiasis surgery | Burma | 107 | 126 |
| Baltussen 2005 | Trichiasis surgery | Americas | 68 | 80 |
| Baltussen 2005 | Trichiasis surgery | Mediterranean | 77 | 91 |
| Baltussen 2005 | Trichiasis surgery | Western Pacific | 48 | 57 |
| Baltussen 2012 | Trichiasis surgery | Sub Saharan Africa | 110 | 130 |
| Baltussen 2012 | Trichiasis surgery | Southeast Asia | 445 | 524 |
| Baltussen 2012 | Cataract repair | Sub Saharan Africa | 141 | 166 |
| Baltussen 2012 | Cataract repair | Southeast Asia | 118 | 139 |
| Chen 2012 | Orthopedic Surgery | Nicaragua | 416 | 490 |
| Chen 2012 | Orthopedic Surgery | Nicaragua | 563 | 663 |
| Moon 2012 | Cleft Repair | Vietnam | 74 | 88 |
| Moon 2012 | Cleft Repair | Vietnam | 106 | 125 |
| Rattray 2013 | Plastic Surgery | Cambodia | 103 | 121 |
| Binagwaho 2013 | Circumcision (HIV prevention) | Rwanda | 370 | 436 |
| Binagwaho 2013 | Circumcision (HIV prevention) | Rwanda | 679 | 800 |
| Source: Chung 2022^24^ |  |  | **ICER (2020 USD)** | **ICER (2021 USD)** |
| Alkire 2011 | Cleft Repair | Sub Saharan Africa | 59 | 62 |
| Corlew 2010 | Cleft Repair | Nepal | 127 | 133 |
| Corlew 2010 | Cleft Repair | Nepal | 48 | 50 |
| Magee 2010 | Cleft Repair | Vietnam | 100 | 105 |
| Magee 2010 | Cleft Repair | Nicaragua | 85 | 89 |
| Magee 2010 | Cleft Repair | Kenya | 146 | 153 |
| Magee 2010 | Cleft Repair | Russia | 50 | 52 |
| Rattray 2013 | Cleft Repair | Cambodia | 103 | 108 |
| Poenaru 2016 | Cleft Repair | 83 countries | 179 | 187 |
| Hackenberg 2015 | Cleft Repair | India | 239 | 250 |
| Hackenberg 2015 | Cleft Repair | India | 440 | 461 |
| Hamze 2017 | Cleft Repair | Eastern/Central Africa | 86 | 90 |
| Nadoskar 202 | Cleft Repair |  | 704 | 737 |
| Source: Chao 2014^25^ |  |  | **ICER (2012 USD)** | **ICER (2021 USD)** |
|  | Cesarean Section (lower bound [LB]) |  | 401 | 478 |
|  | Cesarean Section (upper bound [UB]) |  | 268 | 319 |
|  | Aspirin and beta blocker therapy (LB) |  | 500 | 596 |
|  | Aspirin and beta blocker therapy (UB) |  | 707 | 842 |
|  | Antiretroviral HIV therapy (LB) |  | 454 | 541 |
|  | Antiretroviral HIV therapy (UB) |  | 648 | 772 |
|  | BCG vaccine (LB) |  | 52 | 62 |
|  | BCG vaccine (UB) |  | 220 | 262 |
|  | Other Vaccines (LB) |  | 13 | 15 |
|  | Other Vaccines (UB) |  | 26 | 31 |
|  | Bednets for malaria (LB) |  | 6 | 7 |
|  | Bednets for malaria (UB) |  | 22 | 26 |

**References**

1. Rushby JF. Calculating and presenting disability adjusted life years (DALYs) in cost-effectiveness analysis. *Health Policy Plan*. 2001;16(3):326-331. doi:10.1093/heapol/16.3.326

2. Salomon JA, Haagsma JA, Davis A, et al. Disability weights for the Global Burden of Disease 2013 study. *Lancet Glob Health*. 2015;3(11):e712-e723. doi:10.1016/S2214-109X(15)00069-8

3. Vos T, Lim SS, Abbafati C, et al. Global burden of 369 diseases and injuries in 204 countries and territories, 1990–2019: a systematic analysis for the Global Burden of Disease Study 2019. *The Lancet*. 2020;396(10258):1204-1222. doi:10.1016/S0140-6736(20)30925-9

4. Global Initiative for Children’s Surgery, Seyi-Olajide JO, Anderson JE, et al. Inclusion of Children’s Surgery in National Surgical Plans and Child Health Programmes: the need and roadmap from Global Initiative for Children’s Surgery. *Pediatr Surg Int*. 2021;37(5):529-537. doi:10.1007/s00383-020-04813-x

5. Poenaru D, Pemberton J, Frankfurter C, Cameron BH, Stolk E. Establishing disability weights for congenital pediatric surgical conditions: a multi-modal approach. *Popul Health Metr*. 2017;15(1):8. doi:10.1186/s12963-017-0125-5

6. Yap A, Cheung M, Muzira A, et al. Best Buy in Public Health or Luxury Expense?: The Cost-effectiveness of a Pediatric Operating Room in Uganda From the Societal Perspective. *Ann Surg*. 2021;273(2):379-386. doi:10.1097/SLA.0000000000003263

7. Murray CJL, Aravkin AY, Zheng P, et al. Global burden of 87 risk factors in 204 countries and territories, 1990–2019: a systematic analysis for the Global Burden of Disease Study 2019. *The Lancet*. 2020;396(10258):1223-1249. doi:10.1016/S0140-6736(20)30752-2

8. Ford K, Poenaru D, Moulot O, et al. Gastroschisis: Bellwether for neonatal surgery capacity in low resource settings? *J Pediatr Surg*. 2016;51(8):1262-1267. doi:10.1016/j.jpedsurg.2016.02.090

9. Poenaru D, Pemberton J, Cameron BH. The burden of waiting: DALYs accrued from delayed access to pediatric surgery in Kenya and Canada. *J Pediatr Surg*. 2015;50(5):765-770. doi:10.1016/j.jpedsurg.2015.02.033

10. Ullrich SJ, Kakembo N, Grabski DF, et al. Burden and Outcomes of Neonatal Surgery in Uganda: Results of a Five-Year Prospective Study. *J Surg Res*. 2020;246:93-99. doi:10.1016/j.jss.2019.08.015

11. Shillcutt SD. Cost-effectiveness of Groin Hernia Surgery in the Western Region of Ghana. *Arch Surg*. 2010;145(10):954. doi:10.1001/archsurg.2010.208

12. Eeson G, Birabwa-Male D, Pennington M, Blair GK. Costs and Cost-Effectiveness of Pediatric Inguinal Hernia Repair in Uganda. *World J Surg*. 2015;39(2):343-349. doi:10.1007/s00268-014-2818-2

13. Tran KB, Lang JJ, Compton K, et al. The global burden of cancer attributable to risk factors, 2010–19: a systematic analysis for the Global Burden of Disease Study 2019. *The Lancet*. 2022;400(10352):563-591. doi:10.1016/S0140-6736(22)01438-6

14. Mathers CD, Lopez AD, Murray CJ. The burden of disease and mortality by condition: data, methods, and results for 2001. *Glob Burd Dis Risk Factors*. 2006;45(88):10-1596.

15. Chatterjee S, Gosselin RA. Estimating the effectiveness of a hospital’s interventions in India: impact of the choice of disability weights. *Bull World Health Organ*. 2015;93(7):476-482. doi:10.2471/BLT.14.147900

16. Health Forum. *Estimated Useful Lives of Depreciable Hospital Assets*. American Hospital Association Press; 2018.

17. Biomedical Engineering Advisory Group. *Lifespan of Biomedical Devices*.; 2004. https://www.academia.edu/10481813/Biomedical_Engineering_Advisory_Group_Guidance_Paper_Life_span_of_Biomedical_Devices_Background

18. Sherman JD, Raibley LA, Eckelman MJ. Life Cycle Assessment and Costing Methods for Device Procurement: Comparing Reusable and Single-Use Disposable Laryngoscopes. *Anesth Analg*. 2018;127(2):434-443. doi:10.1213/ANE.0000000000002683

19. Leiden A, Cerdas F, Noriega D, Beyerlein J, Herrmann C. Life cycle assessment of a disposable and a reusable surgery instrument set for spinal fusion surgeries. *Resour Conserv Recycl*. 2020;156:104704. doi:10.1016/j.resconrec.2020.104704

20. McQuerry M, Easter E, Cao A. Disposable versus reusable medical gowns: A performance comparison. *Am J Infect Control*. 2021;49(5):563-570. doi:10.1016/j.ajic.2020.10.013

21. World Health Organization Department of Health Systems Governmence and Financing. WHO-CHOICE estimates of cost for inpatient and outpatient health service delivery. Published online 2010. Accessed May 20, 2023. https://cdn.who.int/media/docs/default-source/health-economics/who-choice-estimates-of-cost-for-inpatient-and-outpatient-health-service-delivery.pdf?sfvrsn=b814d37e_3&download=true

22. WHO-CHOICE. Note on the Methodology used to predict Unit Costs for Patient Services. Published online July 2011. https://cdn.who.int/media/docs/default-source/health-economics/meth_predictunitcps2011.pdf?sfvrsn=ef0cf282_1&download=true

23. Saxton AT, Poenaru D, Ozgediz D, et al. Economic Analysis of Children’s Surgical Care in Low- and Middle-Income Countries: A Systematic Review and Analysis. *PloS One*. 2016;11(10):e0165480-e0165480. doi:10.1371/journal.pone.0165480

24. Chung KY, Ho G, Erman A, Bielecki JM, Forrest CR, Sander B. A Systematic Review of the Cost-Effectiveness of Cleft Care in Low- and Middle-Income Countries: What is Needed? *Cleft Palate Craniofac J*. Published online July 3, 2022:105566562211110. doi:10.1177/10556656221111028

25. Chao TE, Sharma K, Mandigo M, et al. Cost-effectiveness of surgery and its policy implications for global health: a systematic review and analysis. *Lancet Glob Health*. 2014;2(6):e334-e345. doi:10.1016/S2214-109X(14)70213-X
