# Supplementary material for: Cost-effectiveness of a pediatric operating room installation in Sub-Saharan Africa
Source: PLOS Glob Public Health. 2024 Mar 27;4(3):e0001748. doi: 10.1371/journal.pgph.0001748 (PMC10971580; doi:10.1371/journal.pgph.0001748)
Supplement: Attachment — Submitted filename: PLOS Global Health Reviewer Response 20230614.docx [file pgph.0001748.s004.docx]

**Reviewer Comments and Responses**

The authors’ response is written in blue.

**Reviewer #1**

An interesting article. Overall, the intense focus and use of the term “charity/installation perspective” is a bit confusing, calculating DALYs averted just for installing equipment seems a bit irrelevant as that is not how things work. Unless other investments and efforts are made, mainly in terms of personnel, but also others, that equipment would just sit there. It seems that the article grew out of an effort to show a charity the worth of their investment (and maybe inspire other charities to do the same?), but it just seems a bit strange. It is good that the article also includes the full cost perspective. The large added value of having dedicated pediatric ORs is an important point, so I think this is a relevant article.

We thank this reviewer for their insightful comments. The reviewer is right to recognize the proclivity to describe the ‘installation’ perspective as the ‘charity’ perspective. One of the main goals of the charity KidsOR is to install dedicated pediatric surgical infrastructure in low-middle income country (LMIC) sites with a clear lack of operating room space for children for the pre-existing surgical providers. Therefore, we strove to single out the installation arm given the selection of sites for this charity’s model, such that the results can a) evaluate the contributions and return of investment of the charity and b) inform surgical infrastructure building of this sort in similar settings or organizations. To provide more context, we believed that focusing on the installation aspects of the operating room provides information that was immediately actionable, implementable, and scalable for the charity. Currently, KidsOR has 50+ OR installation projects either completed or underway, thus allowing us to project cost-effectiveness in this intervention from the charity perspective would be very informative to potential donors, local stakeholders, and Ministries of Health.

In the introduction, we have added a description of the KidsOR goal to elucidate better how the installation or ‘charity’ cost is important to include: “Charity KidsOR is a non-governmental organization (NGO) with a primary goal to improve the pediatric surgical capacity in LMICs. KidsOR adopts a multifaceted strategy that includes support for pediatric surgical trainees and installation of dedicated pediatric operating rooms (ORs) in LMIC hospitals. In their Africa Action Plan, KidsOR outlined their commitment to install over 100 dedicated pediatric ORs as part of their capacity building efforts by 2030. The intervention was highly cost-effective in the first dedicated pediatric OR installation in Uganda, but the results have not been validated or replicated elsewhere. Here we report a CEA of two dedicated pediatric OR installations adding to a pre-existing pediatric surgical service in Nigeria in a hospital that did not previously have dedicated pediatric OR space.”

The methodology overall seems sound. I have some questions regarding the costs included – it seems unclear, for instance, whether there were additional personnel costs, etc. - see in detailed comments attached.

Thank you for bringing up the issue of personnel costs. We explain our methodology in response to one of your more detailed comments below (in the subheading titled “Page 10, Cost inputs second paragraph”

*Sentences in Italic are quotes from article.*

**Page 5, Abstract:**

The abstract is not very clear and contains some confusing or not very finely honed sentences.

In particular:

3^rd^ sentence contains both *“****Installing and maintaining*** ***a*** *pediatric operating room”and “****installing*** ***two*** *dedicated pediatric operating rooms”?*

The analysis seems to be for the 2 rooms. And the charity view analysis seems to be just for installing, not maintaining or operating.

Thank you for pointing out this misrepresentation of the action and the number of interventions applied. We agree that we are evaluating the ‘installation’ of the pediatric operating room, and therefore have now removed the “and running” phrases and added that “two operating rooms” were installed.

*“Presented from a charity and societal perspective…*”

Usually the main stakeholders would be the agency operating the program (health care systems perspective) and the user (user perspective). All together would present the societal perspective. It sounds as if the hospital costs of staffing and running the OR was only included in the societal perspective. To look at the cost of the installation by the charity without looking at the operating costs incurred by the hospital just seems odd. The cost results presented in the abstracts only refer to the installation, even when referring to the societal cost.

Thank you for this observation. We agree that we have focused on the charity costs specifically to highlight the charity's actions, i.e., the cost of installing two pediatric operating rooms. In further discussion with our health economist, we decided that the “societal perspective” is no longer an accurate characterization of the scope of our CEA model. While indirect medical costs (such as transportation, accommodation, and childcare expenses) were included in our prior analysis, we have not enumerated the opportunity costs (e.g. of lost wages from the patient perspective), which is another important component of the societal perspective. Therefore, to keep our terminology accurate, the term we now use to describe the scope of our CEA model is “all healthcare payor’s perspective”, and have reflected this in our manuscript.

To further clarify the stakeholders that comprise the perspective, we have incorporated descriptions of 3 cost categories: the installation capital costs (which are also the charity costs), the local Ministry of Health (MoH) costs, and the patient out-of-pocket (OOP) perspective. As there is no consistent insurance scheme in Nigeria, patient families generally have to pay for all postoperative medications, diagnostics, and other medical services or supplies. In an additional Table 2 (shown below), we have included the components of each cost category for easier reference of the items that are included in the model. Furthermore, we have demonstrated the breakdown of these categories in our base case analysis with a pie chart to showcase how each perspective contributes to the overall intervention costs (also shown below).

Table 2: The breakdown of installation costs of two pediatric operating rooms by stakeholder, i.e. the local Ministry of Health (MoH), the installation capital (charity), and the patient out-of-pocket (OOP) expenses.

| Local Ministry of Health (MoH) | Personnel, Perioperative costs (surgical and anesthetic consumable equipment, perioperative medications, perioperative utilities), hospitalization inpatient stay |
| --- | --- |
| Installation capital (Charity) | Freight and installation, long-term, large-scale, durable surgical and anesthetic equipment, overhead administrative costs |
| Patient out-of-pocket (OOP) | Family OOP spending for postoperative medications, diagnostics, and other medical services needed for the surgery |

It is mentioned that the hospital already provided pediatric surgical services, but one of the key points of this article is the value of the addition of ORs **dedicated** to pediatric surgery, so the phrase “dedicated pediatric OR” should be included in the abstract and not only on page 10 or wherever it first is mentioned.

Thank you for suggesting this specifier to describe the OR installations. We agree that we need to clarify the type of OR space we were creating for a pre-existing pediatric surgical team, and therefore have changed the description of the OR installation to “dedicated pediatric ORs” in the abstract and elsewhere in body of the manuscript when the intervention is mentioned.

*“ICER of OR installation from the societal perspective*…”

Again, the term installation throughout the article is confusing – Especially when talking about it from the societal perspective, shouldn’t this be installation **and operation** of the two dedicated OR rooms?

Thank you again for bringing this intervention detail to our attention. It is true that we are studying both the installation and the operation of two dedicated pediatric ORs. Therefore, we have added this specification to the phrase when we are describing the intervention. We also want to reiterate that we are no longer using the term “societal perspective”, but rather will opt for the “all healthcare payor’s perspective”.

Later in the article, the authors clarify that the hospital did not have any DEDICATED pediatric ORs.
I think this an important point that should be included in the abstract. The way the abstract is currently phrased makes it sound as if there were just two extra pediatric ORs added.

Similar to our response to this reviewer’s mention of accurately describing the intervention we are modeling; we agree that the specification of “dedicated” pediatric operating rooms should be emphasized and have added these descriptors whenever we describe the intervention. To help clarify this descriptor, have included the definition in the Methods section under the subheading “Study Setting and Participants”, stating: “A dedicated pediatric OR is defined as a designated operating space exclusively for children (and not shared by adult services) that is fully equipped with the necessary anesthetic and surgical instruments and devices designed for pediatric surgeries.”

**Page 7, first paragraph**

Again, I would add the phrase “that had no dedicated pediatric ORs before”

Thank you again for this suggestion to add a clarifying statement to the study settings. We have added an additional clause to showcase the scenario before without a dedicated OR in our study setting. The end of the introduction now reads “Here we report a CEA of the installation of two dedicated pediatric ORs to a pre-existing pediatric surgical service in Nigeria in a hospital that did not previously have dedicated pediatric OR space.” while the beginning of the methods section in the *Study Setting and Participants* now reads “Facilities included a neonatal unit and pediatric surgery service staffed by three accredited pediatric surgeons but initially had no dedicated pediatric ORs.”

**Page 7, second paragraph**

Problem throughout the article - inconsistent tenses – switching between present and past – everything should be in the past

Thank you for bringing up this issue of tenses and for recommending that the past tense to be used consistently. We have rephrased this paragraph to better reflect the consistent past tense voice: “The study was set in a 450-bed national referral hospital in Nigeria that accepted patients from across the country and in the West SSA region. The study site is a government hospital that accepts predominantly indigent patients in a low-income country with little to no health insurance, therefore necessitating substantial out-of-pocket medical costs. Facilities included a neonatal unit and pediatric surgery service staffed by three accredited pediatric surgeons but initially had no dedicated pediatric ORs. A dedicated pediatric OR is defined as a designated operating space exclusively for children (and not shared by adult services) that is fully equipped with the necessary anesthetic and surgical instruments and devices designed for pediatric surgeries. In August 2019, two dedicated pediatric operating rooms were installed philanthropically within the hospital grounds to bolster its pediatric surgical infrastructure and alleviate the surgical backlog. Since then, the pediatric surgical volume and case complexity had significantly increased. This CEA incorporated this increased surgical volume, as additional disease burden was averted after the installation of the pediatric OR.”

**Page 8, Table 1, first sentence**

Should be all the way at the end of the table, not as first assumption

Thank you for pointing out the need to revise the order of assumptions. In fact, we removed this assumption from the table as another reviewer correctly pointed out that it is not valid to assume that length of stay (LOS) costs were not included for patients who had died. Therefore, in our new model analysis, we have included LOS costs and subsequently removed this assumption from our list.

**Page 8, Table 1, last sentence:**

*“seldom fluctuates dramatically”* should be replaced with “is not assumed to fluctuate…”

Thank you for correcting the word choice and phrasing. We have changed this assumption to read as this reviewer has suggested and the language is now made clearer.

**Page 9, Table 1, second -to-last sentence:**

*“The clinical characteristics…”* – rewrite, unclear what is meant

Thank you for asking for more details on the clinical characteristics. We have added an extra clause in brackets to describe the patients’ clinical characteristics to mean presenting age, diagnoses, and survival outcomes. The bullet point now reads: “Patient characteristics (i.e. the patients’ age at time of surgery, diagnosis, and postoperative survival rates) inform the estimates of effectiveness.”

**Page 9, last paragraph:**

“Health effectiveness was informed…” – maybe replace with “Health outcomes/Effectiveness of the additional surgeries”

Thank you for this suggestion to clarify what is meant by health ‘effectiveness’, as we were looking into health outcomes. We have since added this description to the sentence as recommended.

**Page 10, Cost inputs second paragraph**

Clarify – there were no additional salary costs – doctors just performed more operations in the same time? If yes, that is not a sustainable model. Provide a bit more detail.

Thank you for recognizing the nuances of this salary model. We agree that our model would be unsustainable if we did not account for the increased staff workload required to handle more operations. We did mention that annual salary costs were included in the cost inputs of the model and the inclusion of salaries was weighted by the proportion of cases that each staff member was present in. We expounded on this approach in this paragraph, which now states “Personnel costs included the annual salary costs of perioperative staff, which were informed by public salary scales and verified by the perioperative staff employed at the hospital. The personnel costs attributable to the incremental cases after the installation of the ORs were included in the intervention arm of the model, weighted by the proportion of each OR staff member’s presence per case. The presence of OR staff members in surgeries was informed by the perioperative patient registry, which documents the type and number of OR staff members who participated in each case. A detailed explanation of the personnel cost calculations can be found in the Supplementary Materials, under Table S5.”

In the supplemental materials, we added the step-by-step approach in which we calculated the personnel costs and the sources used to determine these costs, which were as follows:

- OR staff member presence in cases: This was collected using the perioperative REDCap patient registry, which provides data on who was present at each case. Therefore, for the extent of the study period, we had case-specific information on which OR staff members covered the case. For example, the perioperative registry informed us that a pediatric anesthesiologist was present in 7.8% of all cases within the study period. To make this proportion applicable to capturing just the “incremental” cases that were performed in the interventional arm, we multiplied the staff member’s % of case presence by the proportion of additional cases that was performed after OR installation. In the base case analysis, this was 34.3%. Therefore, the % of case presence that was attributable to the incremental cases for a pediatric anesthesiologist was 7.8% * 34.3% = 2.7%.
- Staff member salary scales: these were informed by publicly available salary scales set by the Nigerian government and corroborated by the OR staff members at the study hospital. As the salary scales are typically reported in ranges, the midway point was used as the base rate. Using the same example, the pediatric anesthesiologist’s average annual salary in 2021 USD was 45,747.
- The personnel salary attributable to the increased caseload: We calculated this number by multiplying each OR staff member’s annual salary by their proportion of case presence in the incremental number of cases attributable after OR installation. Therefore, following the example of the pediatric anesthesiologist, the attributable salary for the number of cases performed was 45,747 * 2.7% = 1,682.
- Probabilistic sensitivity analysis: The salary scales upper and lower limits were used as the upper and lower bounds of the (gamma) probability distribution. This was the uncertainty factor that we randomized during the Monte Carlo simulation. The other parameters that went into calculating the salary scales stayed constant.

We also wanted to note in contrast to the “fee-for-service” of the private healthcare system, the surgical staff at our study site were on fixed government salary scales, which did not consider per diem hours worked (or the number of operations performed). In fact, surgical backlogs and lack of OR infrastructure meant that even if surgical personnel were available, they would not be able to perform these cases.

**Page 10, Cost inputs last paragraph**

Use of WHO – CHOICE hospital length of stay costs: The WHO estimates include personnel, capital and food costs. First, would there not be some overlap with the other costs included here? Secondly, all the other data in the study were collected locally, it seems strange there was no way to calculate this from hospital records instead of this very generic estimate that was potentially generated through regression analysis of other country data?

Thank you for pointing out that the WHO-CHOICE tool is at a lower resolution of cost estimation compared to our ‘micro-costing’ of the case-specific aspect of running the pediatric ORs. Our perioperative REDCap registry only captured detailed information surrounding perioperative events, and therefore we were able to pay much closer attention to the number of resources utilized around a surgery. The specific resource utilization in-hospital stay afterward was captured in the REDCap registry as the OOP costs reported by patient families, who usually paid for the bulk of the postoperative treatment needs (i.e. medications, tests) given the lack of health insurance. WHO-CHOICE costs include only boarding and overhead costs, but not the treatment costs of the individual patient. (The methodology was found in this document: <https://cdn.who.int/media/docs/default-source/health-economics/meth_predictunitcps2011.pdf?sfvrsn=ef0cf282_1&download=true>. In this methods paper, the in-patient costs include “only the "hotel" component of hospital costs, i.e., excluding the cost of drugs and diagnostic tests”. Therefore, we opted to rely on the relatively blunt approach of the WHO-CHOICE average of the cost of a hospital day per day for the baseline boarding costs. Furthermore, if overlap were to exist, the cost-effectiveness ratio is underestimated and therefore a more conservative measure, such that any finding of cost-effectiveness would be more convincing.

**Page 11, second paragraph**

Should be *how much the ICER change****d****, Ranges* ***were*** *informed,* etc. Tense consistency

Thank you again for pointing out these inconsistencies in tense. We have made the necessary changes to the past tense in this sentence as this reviewer had suggested.

**Page 12, last paragraph**

Age discounting is widely seen as controversial and WHO now discourages its use. In this case it seems that the authors used it mainly to increase the number of DALYs, not sure this adds much.

Thank you for bringing this archaic conversion to our attention. We acknowledge that age weighting can be skewed and agree with the WHO that implementing this adjustment can bias our results. We therefore realize that age weighting is no longer necessary to report and thus have removed mention of this approach from the methodology and findings of the manuscript report.

**Reviewer #2**

This is an extremely important study and very methodologically rigorous. I applaud the authors for their exceptional work. In brief, in this study, the authors describe the cost-effectiveness of installing a dedicated pediatric OR in a Nigerian hospital with a pre-existing pediatric surgical service. Both costs and patient data generating DALYs calculations were empirically driven rather than projected based on epidemiological assumptions, and a notable strength was the inclusion of supplies like reusable equipment, physical OR installation, and patient out-of-pocket costs. This study demonstrates that operating rooms are resources that can utilize the same personnel to accommodate a broad spectrum of surgical needs, reflecting return on investment far beyond single condition cost analyses, as the authors point out using the example of previous cost-benefit studies for cleft lip/palate repair.

Methods

Model assumptions were reasonable and justifiable. Multidimensional sensitivity analyses demonstrated the robustness of the ICER estimates and using real clinical outcomes permitted as accurate a DALYs averted calculation as possible.

In the counterfactual section, the authors report:

“Without a dedicated pediatric OR, only a proportion of patients underwent surgery (66% of the post-installation volume) given insufficient surgical infrastructure.”

This is confusing. Can the authors report actual values generating the percentages? And would it not be clearer to report the percentage stated as the “pre-installation volume” as that is when the measurement occurred (understanding this historical scenario is being used as counterfactual in present scenario)? I think it is the wording at “post-installation volume” that is the cause of confusion.

Thank you for pointing out this confusing report of the comparison of surgical volumes between pre- and post-installation periods. The authors agree that describing the actual surgical volume would be much clearer in conjunction with reporting percentage changes. 226 cases occurred in the year prior to the installation of the ORs, while 343 cases occurred in the year after installation. Therefore, we have reworded this section regarding the counterfactual (or better phrased, “comparator” scenario) as follows:

“The comparator scenario represented the number and case mix of surgeries performed by the pediatric surgical service during the pre-installation phase. The surgical volume was 226 a year prior to the installation of the two dedicated pediatric ORs and increased to 343 cases in the year immediately afterward. Therefore, the pre-installation case volume was 66% (226/343) of the post-installation surgical volume). In the pre-installation phase, 34% of patients who could have otherwise undergone surgery if resources were available instead suffered the natural course of the disease. In these low-resource settings, there is no viable non-surgical alternatives for these children with anatomical defects requiring definitive, mechanical repair. For example, intestinal atresia must be repaired with surgery given the intestinal blockage. An imperforate anus can only be cured with the creation of a new anal canal via surgery. Therefore, without surgery, the natural course of disease is assumed.”

Time discounting and age weighting are appropriate. Can the authors cite the Fox-Rushby et al study supporting their age weighting statement that they make reference in the text to? Are they referring to: Fox-Rushby J, Hanson K. Calculating and presenting disability adjusted life years (DALYs) in cost-effectiveness analysis. Health Policy Plan. 2001;16(3):326–331?

Absolutely - thank you for pointing out our omission of this necessary reference. We have since included this reference in the appropriate location where we mention the Fox-Rushby et al paper.

When the authors say “charity perspective” can they define whether this means from the philanthropic (operational?) perspective of the NGO that supported the equipping of the pediatric ORs, as that becomes the reader’s assumption? Explicit clarification would be helpful, as the “societal perspective” is better defined to include “hospital system, charity, and patients’ families.” Can the authors clarify how the ICERs from both perspectives should be interpreted in context of one another (independently or combined). Later in the discussion the authors state, “This societal perspective reflects the reality of a charity-sponsored public health intervention, recognizing that OR installation does not occur in a vacuum and that local surgical support is necessary” however, expounding upon this in the methods would be helpful.

Thank you for bringing up this important distinction among perspectives that we present in this analysis. The first reviewer had also brought up the confusion of the “charity perspective” vs the “societal perspective”. In this sense, we have decided to omit the mention of an ICER from the “charity perspective” altogether, and also remove the mention of a “societal perspective”. Instead, we decided to focus on one ICER/perspective: the “all healthcare payor’s perspective”. More details on how we have elaborated on this in the methods can be found in our response to reviewer 1’s commentary after their quote *“Presented from a charity and societal perspective…*”. (Page R2).

Results

Relatedly, “The total annualized cost of installation and maintenance of the pediatric operating rooms was $174,114 from the societal perspective or $ 87,728 from the charity perspective. The charity-funded costs made up 50% of the total cost of the intervention.” This is confusing - are you suggesting the total was $261K? Using “or” makes it seem that these were alternatives, while the sentence about charity-funded costs making up 50% of total cost would suggest it’s combined? Please clarify.

Thank you for the request to clarify our main findings in the cost evaluation. We agree that the number of operating rooms makes this report confusing, especially with the inappropriate use of the “or” conjunction. We meant to convey that the total cost of installing and running two dedicated pediatric ORs was $174,114 from the societal perspective (which includes the local health system, the installation, and the patient perspective). Therefore, we reworded the cost findings to: “The total annualized cost of installation and operation of two dedicated pediatric ORs was $177,527 from the all healthcare payor’s perspective. Installation capital (charity) costs made up 50% of the total costs ($ 87,728), while the local MoH costs covered 41% ($72,980) and patient OOP costs made up 6%.”

When the authors report “246 (23%) cases were performed before installation of the additional pediatric ORs (June 2018 - Aug 2019), whereas 822 (77%) cases were performed postinstallation (Aug 2019 - Sept 2021)” can they also add a mean case/month to better represent the data? For instance, over 14 months and 25 months, respectively, cases are 246cases/14months=17.5 and 822cases/25months=32.88, which would seem to better represent the increase in operative capacity post-implementation for readers.

Thank you for this helpful suggestion for adding the monthly average case count to provide a better sense of the surgical volume before and after the installation of the two pediatric ORs. To follow your calculation suggestions, we verified that the case averages were 18.9 cases per month (246 / 13 months) and 32.9 cases per month (882/25 months). “246 (23%) cases were performed before the installation of the additional dedicated pediatric ORs (June 2018 - July 2019), or an average of 18.9 cases per month. A total of 822 (77%) cases were performed post-installation (Aug 2019 - Sept 2021), or an average of 32.9 cases per month.”

In the second paragraph of the results section, after the first sentence about annualized cost of equipment for “two ORs,” please clarify explicitly if the additional sentences also refer to costs for both ORs and if those costs were distributed evenly between ORs for readers.

Thank you for this important need to distinguish whether we are reporting the findings of one or two ORs. For all the results, we were specifically referring to the installation of two dedicated pediatric ORs. Therefore, the revised paragraph now reads as: “From the installation capital (charity) perspective, the annualized cost of purchasing reusable surgical and anesthetic equipment for two dedicated pediatric ORs was £37,953 in 2019 British pounds or $57,435. (Fig 1) The cost of shipping and installation was £19,500 in 2019 British pounds or $30,293.”

Can the authors speak to global supply chain, as they mention the annualized cost of shipping and installation was £19,500 in 2019 uninflated British pounds or $30,293 (vs donated reusable equipment for two ORs being £37,953 in 2019 uninflated British pounds or $57,435). It is significant, and I wonder if the authors could make any comment on feasibility/sustainability of shipping costs.

Thank you for pointing out this current method of shipping international supplies. We acknowledge that the international shipment contributes to a substantial fraction of the overall costs of installation. In fact, shipping costs are a large part of the charity’s expenditures. However, bulk shipments are more cost-effective. KidsOR has actively reduced the number of shipping containers by making pallet packing more efficient as well as environmentally friendly. As KidsOR now have installations due for several ORs for different hospitals in the same country (or neighboring countries), they are paying for one shipment at the same time instead of separate ones. KidsOR sustains excellent relationships with its shipping partners, which helps make it a more sustainable and cost-efficient process.

We have added this context in our Discussion section, stating: “Furthermore, no major surgical equipment suppliers exist in the African continent, and so even if local organizations were invested in building the infrastructure for pediatric surgical ORs, they would still need to internationally ship this specialist equipment. Hopefully this changes in the future, and as KidsOR expands, there are provisional plans for the potential of distribution centers in the continents that the organization is primarily working in.” This, however, is in the far future currently.

In this paper, the authors describe the cost-effectiveness of implementing a dedicated pediatric OR with a pre-existing pediatric surgical service. In many cases where an OR and general surgeons exist, there is no dedicated pediatric surgical service. It would be interesting to hear by how much the authors believe the cost-effectiveness might change if surgeon training was incorporated alongside additional dedicated OR installation, and what projected costs might be like and whether it would remain a feasible endeavor.

We thank this reviewer’s suggestion to incorporate the costs of training a pediatric surgeon, as we agree that more operative space provides the opportunity for a fellowship training program to flourish. In fact, have accounted for this scenario in our model already, as this was the reality at the Abuja National Hospital, which serves as a training site for pediatric surgical fellows. In our personnel costs, we have included the cost of a pediatric surgery fellow, who frequently participates in surgeries and often may be the primary surgeon. These costs are presented in our supplementary materials.

For additional context at Abuja National Hospital, it takes a minimum of 3 years to complete training in pediatric surgery. The accreditation requirement for training needs a minimum of 2 pediatric surgeons, one of whom must be at least 5-years post-training (aka. pediatric surgeon). Per the local team’s report, the most recent annual salaries are estimated to be ~$7,558 for a pediatric surgery trainee is ~$11,270 for a junior pediatric surgeon, and ~$15,686 for a senior pediatric surgeon (in USD EXR). Notably, these numbers are significantly lower than the annual salaries used in the CEA model, as we used the USD PPP as our exchange rates when converting our salaries from the Nigerian Naira, providing a more conservative estimate of our personnel costs and therefore a more conservative ICER.

Hypothetically, if the service to scale up and include another pediatric surgeon, the cost-effectiveness may not change substantially as the annual salary of a pediatric surgical trainee is a small proportion of the overall incremental cost of the intervention. Additionally, the changes in the cost-effectiveness with an additional surgeon may vary case-by-case, and will be due to a balance of operating space, staff, and demand. For example, if another pediatric surgeon joins the service but there is not enough operating room to staff their cases, then the cost-effectiveness will be lower. Conversely, if the pediatric surgeon joins and can operate full-time alongside the other surgeons, then the cost-effectiveness may be higher (as the disease burden averted may surpass the incremental cost of hiring another surgeon).

In the discussion, the authors state “This study’s cost-effectiveness is higher than that of another CEA we conducted for an OR installation in Uganda, at $37·35 per DALY averted.” They should be careful with their wording, as yes their ratio is “higher” (more costly -> higher $ value at $77 and $152), but their reported intervention is actually “less” cost-effective by $/DALY averted (though no less significant), as it costs more to avert each DALY. All this to say, their use of “higher” makes it appear like they are saying the overall CE is higher, when in fact they should be saying their cost is higher, thus making it less cost effective in comparison.

Thank you for pointing out that we may have misconstrued the meaning of the comparative cost-effectiveness of these two studies. “Higher” certainly does not mean “less cost-effective” Therefore, we have rephrased this statement to now read: “The ICER derived for the current study indicated less favorable cost-effectiveness than that of another CEA we conducted for the installation of a single dedicated pediatric OR in Uganda, at $94 (in 2021 USD) per DALY averted.”

The authors mention reduced wait times, improved staff morale, which are major benefits of installing the OR. Can the author discuss how this might be incorporated in future CE indices?

The authors thank this reviewer for this interesting point of mentioning several other benefits that may result from the installation of dedicated pediatric ORs. We believe that the health outcomes from the reduced waitlist were captured by the incremental cases that occurred because of the establishment of two operating rooms. As far as future indices, a potential metric for future economic analysis could be the number of cases in the surgical backlog that was eliminated after the ORs’ installation. The cost-effectiveness ratio could therefore be the cost of eliminating one patient from the list of surgical backlogs. We have included this possibility in our Discussion section, which now reads: “Enhanced capacity meant time delays in surgery were greatly reduced, effectively eliminating wait times that previously extended to years. This was also true for emergency surgeries, where protracted wait times led to more morbidity. The consequences of a long surgical waitlist can range from families’ frustration to patient fatalities. Therefore, from the economic lens, a future potential cost-effectiveness metric could measure the cost of removing a patient from the waiting list as a proxy for improved health outcomes, demonstrating the importance of tracking surgical backlogs as part of a surgical system’s performance. Additionally, since pediatric operating instruments were provided as part of the installation package, surgeries could be conducted with reduced operating times given ready access to the appropriate tools and may be associated with greater staff satisfaction due to having necessary instruments more available.”

The improved staff morale is less easily quantified and therefore we opted to perform a qualitative methods study to interview perioperative staff about their experiences before and after the OR installation. A manuscript of the current qualitative analysis is currently underway.

Limitations are appropriate.

**Reviewer #3:** Pediatric surgery review

General comments

Overall, this paper is well written, thoughtfully analyzed, and addresses an important topic. A few items deserve special commendation. It is commendable that the paper follows the CHEERS guidelines and the authors make their assumptions clear and explicit. The availability of some data from the pre-installation period for comparison with the post period is another important strength of this paper. Most of the analyses appear to be well done and well described. The paper has the potential to make a valuable contribution to the literature.

However, there were a few important instances where it was not clear whether the analysis had been done appropriately. This reviewer feels these items need clarification at a minimum and possibly revision and/or sensitivity analyses. The first concerns the labor and general hospital infrastructure for all aspects of surgery and recovery (such as surgical intensive care units) required for general surgery. The WHO Choice costs count only routine hotel costs of hospitalization. All other costs vary by the type of service and need to be costed separately. In this paper, amortization of equipment and costs of consumable supplies were appropriately described. However, it was not clear how the costs of all personnel (e.g. surgeons, nurses, and technicians) needed in the operating room, pre-operative diagnosis and post-operative care was included. Also, the use and cost of all lab services was not clear. Similarly, the diagnostic and referral processes to identify children with surgical problems and arrange their care was not described and it was not clear whether it had been costed.

Thank you for highlighting a nuanced consideration of the cost inputs for the surgical infrastructure. We strove to address these concerns item by item:

- General hospital infrastructure costs: While we agree that surgical intensive care units would be charged at a higher rate per day of hospital stay and that the WHO CHOICE does not cover these service-specific charges, hospital charges actually do not vary substantially given the lack of specialized equipment (e.g. lack of ventilators, incubators, parenteral nutrition, etc.) in this low-resource hospital setting. Furthermore, there is no pediatric intensive care unit in the hospital at all. The neonatal unit does not have intensive care equipment and facilities. There is an adult intensive care unit that is not equipped to care for children. Therefore, utilization of resources does not differ substantially among patients despite the severity or acuity of their disease, especially for neonates and infants. Unfortunately, due to resource constraints, a neonate freshly out from a complex tracheoesophageal repair does not have vastly different resource utilization from a child after completing an open appendectomy. To account for variation in hospital stay, we have included a probabilistic distribution of a range of hospital costs in our sensitivity analysis to account for this uncertainty.
- Costs of personnel needed in the operating room: We agree that the costs of personnel were not made clear in the Methods. Reviewer 1 had posed a similar question, which we have answered in greater detail under the heading of “Page 10, Cost inputs second paragraph” (Page R5-6)
- Preoperative costs of laboratory and diagnostic services: We agree that patients would need further work-up for their diagnosis and preoperative preparation, but this would happen regardless of whether adequate OR space or surgical capacity exists. Patients will undertake all the necessary tests just so they can be eligible for surgery, but this does not mean they will ultimately receive surgery, due to inadequate surgical capacity leading to surgical backlogs.
- Postoperative costs of laboratory and diagnostic tests, medications, and supplies: These costs are already captured in the OOP expenses reported by patients. As there is no public insurance coverage for patients, families generally must spend money on postsurgical medical supplies. Therefore, we have largely accounted for these treatment costs by virtue of accounting for OOP spending.

The underlying infrastructure was another gap. Did the hospital already have vacant, suitably renovated space for these operating rooms with all the supporting infrastructure, including electricity, back up power, water, laundry, security, administration, etc.? A full economic analysis, generalizable to other settings, should include the economic cost of creating and maintaining such infrastructure.

Thank you for bringing up this important point about supporting infrastructure. For the base case study, we did not include underlying infrastructure cost as the hospital, as the hospital already had a vacant and renovated space for the operating room along with supporting infrastructure. In this scenario, the notion of finding a premade open space is generalizable because this is the model that charity KidsOR chooses to install ORs – in a pre-existing surgical service and hospital with available “dead” space. The reality is that there are typically sunk costs and unused space in these hospital settings, so the purpose of this study is to look at the refurbishment of these empty, otherwise unused spaces.

For context, the study site’s underlying infrastructure in the pre-installation phase can explain why the dedicated pediatric specialist OR was needed:

1. The hospital is a general referral center and pediatric surgery has to compete for limited available resources.
2. There is no dedicated operating room for any specialty.
3. Pediatric surgery was not available when the hospital was established, but instead began much later. As a result, it did not benefit from the initial allocation of operating space. Even when the specialty became available, there was only one pediatric surgeon prior to installation.
4. Although more operating rooms are desired, the hospital did not have the resources to construct and equip new operating rooms.
5. There was an open space in which dedicated pediatric ORs were constructed.

In the case of Abuja National Hospital, the Chief Medical Director/Chief Executive Officer (CEO) for the hospital and the hospital administration were fully in support of the project. The CEO briefed the Minister of Health to put him in the picture beforehand. The hospital already had a well-established theatre fund and theatre users’ committee, which ensures an uninterrupted supply of disposables/reusables and other consumables to the theatres. The arrangement was extended to the installation project. There was also a well-established central sterile supply department, which was fully functional and reliable pre-install.

In terms of utilities, it is also difficult to estimate electricity, water, laundry, and administration costs of the operating rooms as these are part of the existing general hospital resources which were already available and in use before installation of the operating rooms. Nevertheless, we used a reference from Eeson et al. 2015 ([10.1007/s00268-014-2818-2](https://doi.org/10.1007/s00268-014-2818-2)) to estimate utilities used per procedure. In this paper, the average utility cost was $1.80 (2021 USD) per surgery, and so we used this number in our base case analysis. The added cost from perioperative utilities annually is estimated to be $211, which is nominal compared to the overarching cost of the intervention. Nevertheless, this was included in the revised analysis, into the local Ministry of Health cost category.

The paper appropriately notes that there are different versions of DALYs both with and without discounting of future years. A widely accepted text on economic evaluation is MF Drummond et al, Methods for the Economic Evaluation of Health Care Programs. It indicates that health benefits should be discounted. However, the age weighting in the original version of DALYs has since been abandoned. For consistency with current methods, the authors may wish to use discounted life expectancy as a basis for calculating healthy years gained. These would be based on the age of child at the time of surgery, not life expectancy at birth.

Thank you for the helpful citation of the Drummond et al. textbook for our citation. We agree that the age-weighting adjustment is now archaic and have removed this from our analysis. To clarify, our analysis was based on the life expectancy at the time of surgery as this information was available in the perioperative REDCap registry. We have clarified this in our assumptions list. Furthermore, we have redone the analysis to only report 3%-time discounted DALYs, as this is the standard discount rate used across most other CEAs, allowing for more ready comparisons. We had previously omitted the 3% time discount in concordance with the more recent Global Burden of Disease studies to use the “Simplified DALY”, and had reported undiscounted DALYs and ICERs in our previous Ugandan study. However, we understand that this method does not align with the most recent CHEERS guidelines. Therefore, we now exclude any mention of undiscounted DALYs in our revised manuscript, except in our one-way sensitivity analysis. In our ICER comparison figure in the discussion section, we have curated the sources and removed ICERs that were not specified to be time discounted at 3% and made sure that all ICER currencies were reported (inflated) to 2021 US dollars. Therefore, the types of interventions and the ICER ranges changed for some of the reported values. Nevertheless, we still find that the OR installation ICER (ranging from $94-330, based on our current study and the ICER of our prior study in Uganda, which is now time discounted to 3% and reported in 2021 US dollars) is more cost-effective than several other well-known public health interventions.

These, gaps create an important inconsistencies and possible bias. The DALY approach counts the full DALYs for treating additional surgical illness without discounting and assumes that all surgery occurs at birth/ However, the cost analyses, as understood by this reviewer, count only a portion of the real resources used.

Thank you for bringing up these important considerations of the potential inconsistencies in our analysis. We have responded and addressed them to the best of our knowledge and recognize that these changes have made the manuscript and study findings much stronger and more consistent.

Specific comments

Note. The page numbers cited here refer to the automatic page numbers in the pdf file received by this reviewer, which are not necessarily the numbers printed at the bottom of manuscript pages.

In the interest of full disclosure, it would be helpful in the conflict statement to indicate the philanthropy which funded the establishment of the OR and its relation to the funding for the authors.

We thank this reviewer for the ethical guidance to provide full disclosure of the source of funding that KidsOR provided. As such, we have now listed the disclosures as: “Declaration of Interests and Funding Sources: Charity KidsOR funds the installation of the pediatric operating rooms and the data collection process of the perioperative patient data.”

p 6. Despite their wide use, the authors should define LMIC and HIC.

Thank you for pointing out the need to spell out our abbreviations of LMIC and HIC, as the authors that all abbreviations should be properly defined without assuming a prior knowledge of the readers. The LMIC abbreviation is defined in the first sentence of the introduction. We added the definition of HIC in the last sentence of the second paragraph.

P 7. It would seem helpful to distinguish expanding surgical capacity from a pediatric operating room. The authors note that some pediatric surgery has been done successfully without a pediatric OR.

Thank you for providing this distinction between expanding surgical capacity and investing in pediatric surgical infrastructure such as a pediatric OR. We have rephrased this statement to showcase that lack of dedicated pediatric ORs may be a primary reason why there is poor surgical capacity. This section now reads as follows: “Charity KidsOR is a non-governmental organization (NGO) with a primary goal to improve the pediatric surgical capacity in LMICs. KidsOR adopts a multifaceted strategy that includes support for pediatric surgical trainees and the installation of dedicated pediatric operating rooms (ORs) in LMIC hospitals. In their Africa Action Plan, KidsOR outlined their commitment to install over 100 dedicated pediatric ORs as part of their capacity building efforts by 2030.”

P8 While it is commendable that the paper follows the CHEERS guidelines, it would be helpful for the authors to include the CHEERS checklist as a supplemental document to document this.

Thank you for pointing out the necessity for including the actual checklist for ease of interpreting the fulfillment of the CHEERS criteria. We have now included this as an additional attachment to the submission package as supplemental material for readers’ review.

P8 The authors first assumption was: “Patients who pass away before discharge or shortly after surgery do not accrue length-of-stay costs.” As this assumption appears to bias results in favor of the pediatric OR, it deserves explanation. It would seem better to include their actual length of stay.

Thank you for catching this assumption’s inherent bias. We acknowledge that this omission of the length of stay costs for those who had died does bias the ICER’s favorability of the intervention. Therefore, in our updated analysis, we did include this length of stay cost for patients that died. As a result, we have now removed this assumption from our list.

P8 While the availability of some data from the pre-installation period is a strength of this paper, the assumption about the absence of other treatment needs more justification. For example, the web site of Cincinnati Children’s Hospital describes the repair of a condition mentioned by the authors with a high mortality as follows: “With a simple gastroschisis, treatment often is what’s called a ‘primary repair.’ This is a surgery where the bowel is placed back inside of the baby’s belly and the abdominal opening is closed. When possible, this surgery is done the day your baby is born. This type of repair is performed when there’s relatively small amount of bowel outside of the belly, and the bowel is not overly swollen or damaged.” It would be helpful to discuss the applicability of such procedures to the Nigerian context before and after the initiation of the pediatric ORs.

While we agree that there may be temporizing measures that can be provided for children living with pediatric surgical diseases, we do not typically see non-surgical alternatives to diseases caused by anatomic defects that require mechanical repairs. We have elaborated on this reality in the Methods section under the “Comparator” subheading, stating: “In these low-resource settings, there is no viable non-surgical alternatives for these children with anatomical defects requiring definitive, mechanical repair. For example, intestinal atresia must be repaired with surgery given the intestinal blockage. An imperforate anus can only be cured with the creation of a new anal canal via surgery. Therefore, the natural course of disease is assumed.”

Furthermore, most patients at Abuja, across all disease spectrums, present later than in HICs, often with advanced pathologies and physiologic derangements. For gastroschisis specifically, infants usually present with large amounts of eviscerated and edematous intestine, and primary repair is not feasible or safe. These infants, therefore, do not go to the OR but have a silo bag covering the evisceration for gradual reduction before repair of the defect after several days. Installation of the ORs has not changed our approach to treatment. However, installation of the ORs has made it possible for emergent cases and severely ill children to receive surgery faster than prior to OR installation. Additionally, available equipment means that our anesthesiologists are more confident and comfortable with the intraoperative monitoring of such patients. A related study in Uganda demonstrates the alarmingly high mortality in these infants of nearing 100%. (https://doi.org/10.1016/j.jpedsurg.2016.07.011)

P9 The authors note that the life expectancy in Nigeria is 54 years. Presumably, this is at birth. It would be more relevant to give the life expectancy at the average age of the children receiving surgery.

The life expectancy was in fact counted from the time of surgery, such that the remaining life expectancy was considered for each procedure. This information was available to us based on the perioperative REDCap registry, which listed the age of the patient at the time of surgery. Thus, for each operation performed, we calculated the remaining life expectancy by subtracting the average age of the patient(s) at the time of surgery from 54 years. In the assumptions table, we have clarified this aspect by stating: “Life expectancy in Nigeria is 54 years in 2019, per the World Bank, which was used to find the procedure-specific remaining life expectancy by subtracting the average age of the patient(s) at time of surgery from this value.”

P 10 It would be helpful to supply the citation for Fox-Rushby in the text.

Absolutely - thank you for pointing out our omission of this necessary reference. We have since included this reference in the appropriate location where we mention the Fox-Rushby et al paper.

P10 Discussion of the opportunity cost of public sector surgeons would be helpful. If they also have private practices, the time they are able to devote to surgery in the public sector may be limited.

Thank you for this query about the local opportunity costs of practicing in the public sector. As the reviewer surmised, financial compensation in private practice in Nigeria is far more than in the public sector. However, the private sector is unable to employ a pediatric surgeon full-time given the relatively small number of cases requiring pediatric surgical expertise and only invites the pediatric surgeon for specific cases. To put things in perspective, what the pediatric surgeon earns from a single major surgery in private practice is usually about 25 – 50% of the monthly salary in the public sector. Typically, these surgeries can be scheduled around the pediatric surgeons’ days off from the public sector shifts. Therefore, the opportunity cost is negligible given the paucity of cases in the private sector that need coverage from a pediatric surgeon, allowing surgeons to incorporate them into their schedules.

P10 It was not clear how the labor for the surgical procedure itself was calculated. That is not part of the WHO CHOICE costs.

Thank you for bringing this to our attention for the need to elaborate on our methods of calculating labor costs for the surgical procedure. We agree that the current description of how we calculated labor is not clear and we strove to elaborate on our methods in the specific paragraph and in the Supplementary materials. We addressed a similar question posed by Reviewer 1 –see our response under the heading “Page 10, Cost inputs second paragraph”. (Page R5-6)

P11 It would be helpful to clarify where the authors are using US dollars at market exchange rates or PPP dollars. For example, this reviewer found current World Bank per capita GDP for Nigeria in 2021 of $2065 in current USD and $4922.63 in PPP dollars.

Thank you for inquiring about this aspect of the conversion rate. We used PPP dollars, as stated in the first paragraph of the Cost Input subsection of the Methods section. We decided to use the PPP approach as the conversion from resource costs in Nigerian Naira to USD would produce higher monetary values and therefore provide a more conservative measure of the cost-effectiveness ratio.

P12 Table 2 nicely gives the type of distribution. The supplement gives many unit costs, but some still seem to be missing, such as the cost of each hospital day.

Thank you for bringing to our attention this omission of the hospital day costs in the Supplementary materials. We have since added the table of WHO-CHOICE tool values to which we have derived our probability distributions, including the supporting reference and the inflation rate used. We also added a section describing the patient cost perspective and the average costs that informed the out-of-pocket cost input parameters.

P12 The DALYs averted appear to assume that the cases would not otherwise have been treated. Readers would be interested in the percentage distribution of diagnoses and/or surgical procedures in the pre- and post-periods.

Thank you for this insightful comment and request to showcase the pre- and post-installation case distributions. We have reported the changes in the case mix in our prior publication showcasing the case volumes before and after OR installation (https://doi.org/10.1016/j.jpedsurg.2022.09.021). In this report, we found that after OR installation, the number of neonatal surgeries and cases classified as American Society of Anesthesiologists (ASA) class >2 increased significantly, without a concomitant increase in mortality. These findings suggest that more disease burden per patient may have been averted post-installation, given that younger children with more severe diseases were receiving curative surgery. However, we could not include this change in the case mix pre- to post-installation in our model, which was based on the probability of each individual patient receiving surgery. Therefore, the same batch of patients (and therefore the same disease or case mix) would be put through the intervention and comparator arm. We stated this in our limitations: “The ICER’s effectiveness rests on the reliance on the absolute increase in cases after operating room installation, while the increase in patient clinical complexity after OR installation was not included in this study, as our analysis only considered a difference in case volume and assumed the same case mix before and after installation of the ORs. However, this suggests our estimate of the cost-effectiveness is more conservative, as we did not incorporate surgeries of more complex, disabling diseases that may lead to more DALYs averted.”

P12 It would be helpful to supply the reference to the WHO-Choice tool, the data year used, and the approach for inflation adjustment if done.

Thank you for suggesting this important addition to incorporate the specific reference supporting the WHO-CHOICE figures. The authors have cited the report “WHO-CHOICE Estimates of Cost for Inpatient and Outpatient Health Service Delivery” in the Supplementary Material, Table S6. This document was published in 2021 and provides 2010 USD estimates of the “hotel costs” of daily in-hospital stays. In the supplementary materials, we have also started the inflation rate to 2021 USD as 25.46%. The supporting reference we used can be found here: <https://cdn.who.int/media/docs/default-source/health-economics/who-choice-estimates-of-cost-for-inpatient-and-outpatient-health-service-delivery.pdf?sfvrsn=b814d37e_3&download=true>

P13 The wording is unclear whether the cost-effectiveness analysis is comparing the presence of 1 or 2 pediatric ORs against none, or the presence of 2 ORs versus 1 OR.

Thank you for the need to specify the number of OR installations we are comparing to. Unless otherwise mentioned, we have been referring to the intervention arm as the installation and operation of 2 ORs. We have made additional effort to make this reference apparent in our discussion of the intervention. As such, this section of our discussion now reads: “We found that compared to no installation, the installation of two dedicated pediatric ORs in an LMIC hospital with a pre-existing pediatric surgical service is very cost-effective. Utilizing retrospective and prospective data collection, we incorporated real individual clinical outcomes, allowing for a realistic representation of the disease burden averted. Deterministic and probabilistic sensitivity analyses affirmed the robustness of the results. // The ICER from the all healthcare payor’s perspective ($330 per DALY averted) was substantially lower than half of the country’s GDP-per-capita cutoff ($1043 in 2020), which is a cost-effectiveness threshold proposed for LMICs. This study’s ICERs were also compared to that of prior cost-effectiveness studies involving surgeries or other public health initiatives. (Fig 4) The installation of two dedicated pediatric ORs was at least as cost-effective as some other children’s and adult surgery interventions, and more cost-effective than orthopedic surgery, Cesarean sections, medical therapy for cardiac conditions, and antiretroviral therapy for human immunodeficiency virus (HIV), and circumcision for HIV prevention.”

P13 As noted on p 12, it would be helpful to see a table of the most frequent procedures and some type of grouping or categorization for all procedures.

Thank you for this helpful suggestion in categorizing the procedures. We have provided a categorization of the procedure frequencies (along with their DALYs averted) in the bar graph of Figure 2 and have also put the relative distributions of these procedures in a pie chart in the supplementary materials, which we display below. We have also now included a table of the most frequent procedures performed in the supplementary materials, also shown below.

| Procedure | N | % |
| --- | --- | --- |
| Circumcision | 359 | 33.61 |
| Inguinal Hernia Repair | 77 | 7.21 |
| Umbilical/Epigastric Hernia Repair | 42 | 3.93 |
| Appendectomy | 31 | 2.90 |
| Orchiopexy | 30 | 2.81 |
| Hypospadias Reconstruction | 27 | 2.53 |
| Hydrocelectomy | 19 | 1.78 |
| Laparotomy | 17 | 1.59 |
| Intussusception Reduction | 14 | 1.31 |
| Ventriculoperitoneal Shunt | 12 | 1.12 |

P14 Nice to see recent cost-effectiveness thresholds used (refs 18 and 21).

Thank you for acknowledging these references.

P14 The authors report that this hospital already had a pediatric surgical service but lacked a pediatric operating room. This situation is expected to be especially favorable. It counts all the benefits from the pediatric OR but does not count the full cost of establishing the pediatric service and all the diagnostic and referral processes that were likely needed to identify the pediatric cases. The contrast with Uganda assists with this understanding.

Thank you for acknowledging that the OR intervention does not account for the full cost of the pediatric surgical service. Our all healthcare payor’s perspective incorporates some of these costs, but not all. Other surgical CEAs also only partially account for the entire surgical service and referral processes by virtue of being case/procedure-specific. In the time of the KidsOR installation in Uganda, the hospital was currently undergoing renovation and therefore there was no operating space at all despite having a pediatric surgical service.

P17 The authors excluded charity overhead because they were difficult to attribute. Implicitly, this process sets the overhead to zero, which the authors know is inaccurate. A more reasonable process would use a best estimate. In the US, financial data of large charities are public. Administrative costs are around 10%-15% of total costs. That would make them 11% to 17% of direct (program costs). This reviewer feels the analysis would be improved by the authors giving their best estimate of administrative costs and the rationale behind that estimate.

Thank you for this excellent point and suggestion to include overall administrative costs. We believe that this should be a part of the CEA model and have thus incorporated estimated overhead costs based on the charity’s report of the proportion spent. Currently, the charity spends approximately 8% of its funds on administrative costs, and therefore we have built this into our base-case analysis, which amounted to $7,018 for this intervention of installing two pediatric operating rooms. In our one-way sensitivity analysis, we further created a range between 0-16%. We found that the ICER was not sensitive to changes in administrative costs, as it ranged from $317 to $343 per DALY averted in this scenario.
