# Supplementary material for: Cost-effectiveness of a pediatric operating room installation in Sub-Saharan Africa
Source: PLOS Glob Public Health. 2024 Mar 27;4(3):e0001748. doi: 10.1371/journal.pgph.0001748 (PMC10971580; doi:10.1371/journal.pgph.0001748)
Supplement: Attachment — Submitted filename: Response to Reviewers Round 2.docx [file pgph.0001748.s005.docx]

**Response to Reviewers**

Authors’ responses are in blue.

1. Please review your reference list to ensure that it is complete and correct. If you have cited papers that have been retracted, please include the rationale for doing so in the manuscript text, or remove these references and replace them with relevant current references. Any changes to the reference list should be mentioned in the rebuttal letter that accompanies your revised manuscript. If you need to cite a retracted article, indicate the article’s retracted status in the References list and also include a citation and full reference for the retraction notice.

We have reviewed our list of references and did not find any references that have been retracted since the submission of our first version of the article. Therefore, we did not make any alterations to our bibliography.

2. Please amend your detailed Financial Disclosure statement. This is published with the article. It must therefore be completed in full sentences and contain the exact wording you wish to be published.

a. State the initials, alongside each funding source, of each author to receive each grant.
b. State what role the funders took in the study. If the funders had no role in your study, please state: “The funders had no role in study design, data collection and analysis, decision to publish, or preparation of the manuscript.”
c. If any authors received a salary from any of your funders, please state which authors and which funders.

If you did not receive any funding for this study, please simply state: “The authors received no specific funding for this work.”

We have made the necessary amendments to the Financial Disclosure statement, which now reads: “This study was not funded by any grants or commercial funding. Charity KidsOR funded the installation of the operating rooms and the collection of the perioperative patient database. Authors MC, EB, and MF received salaries from the charity KidsOR. The funder KidsOR had no role in the study design, data analysis, preparation of the manuscript, or the decision to publish.”

3. We have noticed that you have a list of Supporting Information legends in your manuscript. However, there are no corresponding files uploaded to the submission. Please upload them as separate files with the item type 'Supporting Information'.

We believed we have uploaded our supplementary files under the “Supporting Information” category appropriately as the editors have requested. Please see the below screenshot as proof of upload:

Reviewer #4: Dear Editor,

I would like to thank the authors for working on this manuscript. It has potential to provide information that has relevance and can inform resource allocation decisions. But it’s apparent that some important information what would make the manuscript both robust and clear are missing. The previous reviewers did mention several good points that have been taken into account during the revision of this manuscript. Nonetheless, I have the following concerns that the authors need to address.

a) The authors have used a decision tree model for the cost-effectiveness analysis. Its good practice to include a schema of such a model showing key decision nodes and chance nodes for example on access or coverage with pediatric surgery and chance/ risk of developing complication after surgery in the main paper. This way any critical reader can immediately appreciate the structure and key components of the model used.

Thank you for pointing out the need to clarify the decision tree model in the body of our manuscript. We agree on the need to move the decision tree diagram from the supplementary materials into the main body of the manuscript for a more central focus of our schema and have made this figure into “Figure 1” of the manuscript.

The model and time horizon section should be improved to reflect this. The decisions are install dedicated pediatric OR and standard of care. For each of these decisions, we need to know at minimum the proportion of children that had access to surgery, the proportion of elective/ emergency surgeries done and complications / recovery rates. The model in the supplimentatary section does not include this key information and I wonder why???

Thank you for this reviewer’s request to elucidate the summary statistics of our patient population and timeframe. Moving forward, we have referred to the two comparator scenarios as “Standard of care” and “Dedicated pediatric OR”.

As for the summary statistics, we have included some of these data points in our results section as we believe these reflected more of the study's findings rather than its methods. We have now added complication/recovery rates as a function of inpatient mortality or survival, which was 1.69% (n=18). We agree that this information is considered key, and therefore, we have included this in the Results section of the main body of the manuscript instead of the supplementary materials. The first paragraph of the Results section now reads: “Over 3.2 years, the pediatric surgical surgery service at the hospital performed a total of 1,068 procedures or a mean of 334 cases per year. 246 (23%) cases were performed before the installation of the additional dedicated pediatric ORs (June 2018 - July 2019), or an average of 18.9 cases per month. A total of 822 (77%) cases were performed post-installation (Aug 2019 - Sept 2021), or an average of 32.9 cases per month. The mean patient age at the time of operation was 3.84 years old (standard deviation [SD] 4.61), and 79.2% (n=846) were male. Of all the cases performed, 806 (75.5%) were elective and 242 (23%) were emergencies. Eighteen patients (1.7%) died after undergoing surgery while inpatient. Notably, in-hospital mortality was significantly higher in those who underwent emergency surgery (16/242, 6.6%) than elective surgery (2/806, 0.2%) using a chi-squared test of difference (p<0.001). General pediatric surgical disease comprised most diagnoses (689, 65%), distantly followed by congenital anomalies (147, 14%).”

As for the “proportion of children that had access to surgery”, we do not know the proportion of the general pediatric population who had access to surgery. The focus of our study group was the population of children who received surgery at Abuja Hospital. By de facto, the proportion of children who had access to surgery in the scenario with the pediatric surgical OR was 100%. By our model design, the proportion of children who had access to surgery without the pediatric OR in place was the number of patients who underwent surgery before installation (numerator) divided by the number of patients who underwent surgery after installation (denominator). We have listed this proportion as 66% in the “Comparator” subsection of the Methods section, stated as: “The surgical volume was 226 a year prior to the installation of the two dedicated pediatric ORs and increased to 343 cases in the year immediately afterward. Therefore, the pre-installation case volume was 66% (226/343) of the post-installation surgical volume). In the pre-installation phase, 34% of patients who could have otherwise undergone surgery if resources were available instead suffered the natural course of the disease.”

b) The section on comparator provides some information about coverage or access to surgical services but I disagree completely with how this is calculated. For the baseline case/ before dedicated pediatric OR, the denominator should be all children that were eligible for surgery. And the numerator is the number of children who actually received surgery (n=226). Similarly for the dedicated pediatric OR period, the denominator should be all children that were eligible for surgery during that period and numerator is the number of the children that received surgical care (n=343) . Even with a dedicated pediatric OR, I cannot imagine all the children that were eligible for surgery were operated, that coverage would not be 100%.

Thank you for the fine attention to detail paid to the calculation of the probability of patients who may or may not be eligible for surgery. We would like to justify the use of our denominator and numerator in calculating the incremental cost-effectiveness as 226 / 343, respectively (aka the two comparator scenarios as stated in the “Comparator” subsection of the Methods section). We clarify that our goal was *not* to calculate the incidence of children who were eligible for surgery who did receive surgery. (See above response to comment “a”). We agree with the reviewer that a single hospital system would not be able to provide surgical access to all children who are eligible to have surgery in the country or catchment area. Our goal was instead to estimate the incremental benefit a dedicated pediatric operating room could offer a pre-existing hospital system with pediatric surgical capabilities, understanding that lack of surgical infrastructure is often the bottleneck for children to access surgical care. If we were to use the reviewer’s reasoning to use the denominator of children who were eligible to surgery, our calculations would still be the same as the children who are eligible for surgery cancel out when we look at the relative incremental effect, provided that we assume that children who are eligible for surgery stay the same over time:

Standard of care	= 226 / [children who are eligible for surgery]

Dedicated pediatric OR	= 343 / [children who are eligible for surgery]

(Relative) incremental effect	= Dedicated pediatric OR / Standard of care

= $\frac{(343/\left[ children who are eligible for surgery \right])}{(226/\left[ children who are eligible for surgery \right])}$

= 343 / 226

To reinforce this assumption, we have added this point explicitly to our list of assumptions, stating that “The prevalence of children who are eligible for surgery within the catchment area of the hospital remains the same over time.”

c) Effectiveness inputs. This section is important but again it misses crucial information. First, note that effectiveness is measure in terms of change/ increase in number or surgeries and (most likely though not shown, reductions in complications due to prompt surgical care). See my comment about importance of having a model schema with key information. How intervention / dedicated pediatric OR effectiveness was ascertained should be clearly described in this section. Second, to calculate DALYs one needs to have information of an ailment e.g., gastroschisis , anorectal malformation, its duration, and disability weight. Important to explain clearly if you estimated the DALYs on a case by case basis at individual level or you averaged or estimated per conditions i.e. all children with surgical condition X were assumed to have had the condition so same duration and thus had same DALYs . Supplementary figure S2 hints on the latter but the write up suggests the former.

Thank you for indicating the need to clarify our methodology in calculating our health effectiveness. We used a combination of individualized outcomes data and disease-specific average health states. We used the hospital’s patient database to inform the incidence of inpatient mortality. For health states other than full cure (e.g. postoperative presence of an ostomy), we used the average disability weights (as informed by previous literature) multiplied by the years of remaining life expectancy. We have showcased our calculations in formulas listed in the Supplementary Material, and have addended our Methods section to indicate this as well. The “Effectiveness Inputs” subsection of the Methods section now states: “We calculated the health outcomes of surgical treatment using disability-adjusted life years (DALYs) and lives saved. In the base-case model, individual inpatient survival was incorporated into the estimation of the health effectiveness. Unless otherwise specified, DALYs averted were subject to a 3% time discount, as recommended by Fox-Rushby et al.(19) Disease-specific disability weights were used to calculate the average health state of those who live in residual disability, which are informed by previously published literature. (Supplementary Materials, Table S1) In our sensitivity analysis, we further subject DALYs to a time discount range between 0-6%. Formulas for the of DALY calculations are showcased in supplementary materials (page 1).”

d) Sensitivity analysis should include key parameters (Re Fig 2: Tornado diagram). One would expect coverage / access, % of emergencies surgeries and complications rates to influence outcomes and these should have been considered during the sensitivity analysis but they have not. I suggest they should and must be reported. If the model did not include these parameters, I would seriously doubt its validity to represent the problem at hand.

Thank you for suggesting the need to include scenarios regarding sensitivity analysis and emergency surgeries/complication rates. Note that we have included the coverage and access as part of the original one-way sensitivity analysis, as depicted in the tornado diagram. In this sensitivity analysis, we varied the “standard of care” scenario to range from 0-90% case volume of the “dedicated pediatric OR” scenario. The resulting ICER was relatively sensitive to changes in this parameter, ranging from $202 to $842 with a range of $640 per DALY averted. To address the need to vary complication rates and elective vs. emergent cases, we added a one-way sensitivity analysis scenario where we adjusted the mortality rate from 0.2% (for elective cases) to 6.6% (for emergent cases). Here, the ICER ranged from $325 to $348, with a difference of $23 per DALY averted, indicating that the ICER was not very sensitive to this adjustment in postoperative complications or emergent/elective caseload. We have revised our tornado diagram to incorporate this additional scenario and added some language describing the results of this one-way sensitivity analysis.

The results describing this analysis now read: “In the one-way univariate sensitivity analysis, the ICER was most sensitive to the proportion of cases that were performed prior to installation of the ORs, ranging from $202-842 per DALY averted when the range of cases was between 0-90% of surgeries that was performed after the ORs’ installation. The ICER was also sensitive to the presence of discounting ranging from 0-6% ($133-434). Conversely, the ICER was relatively insensitive to currency exchange methodology, changes in life expectancy, postoperative mortality rate, cost of reusable equipment, cost of hospital stay, charity administrative costs, and salary.(Fig 2) Note that the mortality range was derived from the difference in mortality between the elective and emergent cases in this hospital.”

e) Its good practice to include a table showing for the study options : costs, incremental costs health outcomes(DALYs) and incremental health outcomes(DALYs) and ICERs in the results section so any keen reader can view and follow your calculations.

Thank you for suggesting a more organized approach to report our findings in tabular format. For the costs, we have reported the breakdown in categories in the pie chart. We agree that showcasing costs and DALYS for each arm of the decision tree and the incremental costs and DALYs of the base case scenario would be helpful. Therefore, we have included a table to depict as such:

**Table 4:** Costs and effectiveness for the two comparator scenarios, with their incremental differences and the incremental cost-effectiveness ratio (ICER).

| *Annually* | Standard of Care | Pediatric OR | Incremental Difference |
| --- | --- | --- | --- |
| DALYs | 840 | 302 | -538 |
| Total Cost | $117,514 | $295,041 | $117,514 |
| ICER |  |  | $330 per DALY averted |

f) Re Fig3: Cost effectiveness plane: This looks weird and hard to understand. You cannot have ICER values before and after dedicated OR. Unless you are showing average costs per DALY averted before and after, which is meaningless anyways, this table so crucial in CEA reports should be revisited.

Thank you for bringing to our attention this element of confusion regarding the temporality of the cost-effectiveness plane. We agree that the terminology of the “before” and “after” scenarios are confusing, so we have changed this to reflect “standard of care” and “dedicated pediatric OR” scenarios instead per this reviewer’s recommendation. As we have mentioned in our response to comment “e”, we have included a table to showcase the results in tabular format. However, we prefer to keep the cost-effectiveness plane to visually showcase the results of the Monte Carlo probabilistic sensitivity analysis. This type of visualization has been described and encouraged in other studies, including Figure 19.4 in the Economic Analysis section of the Disease Control Priorities 3^rd^ Edition: Essential Surgery published by the World Bank. (Shrime, M., Verguet, S., Johansson, K., Desalegn, D., Jamison, D. T. , et. al. 2015. *“Task-Sharing or Public Finance for Expanding Surgical Access in Rural Ethiopia: An Extended Cost-Effectiveness Analysis”*. In: *Disease Control Priorities* (third edition): Volume 1, *Essential Surgery*, edited by H. Debas, P. Donkor, A. Gawande, D. T. Jamison, M. Kruk, C. N. Mock. Washington, DC: World Bank.) https://www.dcp-3.org/sites/default/files/chapters/DCP3_Essential%20Surgery_Ch19.pdf

g) Limitations section. Ideally, effectiveness estimates for CEA should come from randomized studies conducted under routine/ real world settings. The study design used in this study is a before and after, with all its inherent weakness as regards causality. This should be acknowledged.

Thank you for this insightful comment regarding the limitations of our data sources. We recognize that level 1 evidence should be used in CEA should it be available, but we worked under the constraints of a resource- and knowledge-scarce setting without readily available RCT data, which we agree is a limitation. Furthermore, we utilized firsthand, empirical data on the ground to inform our cost and effectiveness parameters, truly drawing from real-world settings. Therefore, we have added this statement to our limitations: “Furthermore, our study design relied on the difference in caseload before and after the OR installation, which limits the inferences of causality. Nevertheless, our model inputs were drawn empirically from primary sources on the ground, such that the findings should still reflect real-world settings.”
