# Supplementary material for: Cost-effectiveness of a pediatric operating room installation in Sub-Saharan Africa
Source: PLOS Glob Public Health. 2024 Mar 27;4(3):e0001748. doi: 10.1371/journal.pgph.0001748 (PMC10971580; doi:10.1371/journal.pgph.0001748)
Supplement: Attachment — Submitted filename: Final comments.docx [file pgph.0001748.s006.docx]

No more comments. The authors have adequately addressed previous comments.
